# Listen to Your Gut: Key Concepts for Bioengineering Advanced Models of the Intestine

**DOI:** 10.1002/advs.202302165

**Published:** 2023-11-27

**Authors:** Oliver Cameron, Joana F. Neves, Eileen Gentleman

**Affiliations:** ^1^ Centre for Craniofacial and Regenerative Biology King's College London London SE1 9RT UK; ^2^ Centre for Host‐Microbiome Interactions King's College London London SE1 9RT UK; ^3^ Department of Biomedical Sciences University of Lausanne Lausanne 1005 Switzerland

**Keywords:** biomaterials, disease modelling, intestine, organoids, organ‐on‐chip

## Abstract

The intestine performs functions central to human health by breaking down food and absorbing nutrients while maintaining a selective barrier against the intestinal microbiome. Key to this barrier function are the combined efforts of lumen‐lining specialized intestinal epithelial cells, and the supportive underlying immune cell‐rich stromal tissue. The discovery that the intestinal epithelium can be reproduced in vitro as intestinal organoids introduced a new way to understand intestinal development, homeostasis, and disease. However, organoids reflect the intestinal epithelium in isolation whereas the underlying tissue also contains myriad cell types and impressive chemical and structural complexity. This review dissects the cellular and matrix components of the intestine and discusses strategies to replicate them in vitro using principles drawing from bottom‐up biological self‐organization and top‐down bioengineering. It also covers the cellular, biochemical and biophysical features of the intestinal microenvironment and how these can be replicated in vitro by combining strategies from organoid biology with materials science. Particularly accessible chemistries that mimic the native extracellular matrix are discussed, and bioengineering approaches that aim to overcome limitations in modelling the intestine are critically evaluated. Finally, the review considers how further advances may extend the applications of intestinal models and their suitability for clinical therapies.

## Graphical abstract

The intestinal epithelium can be reproduced in vitro as intestinal organoids; however, the native intestine also contains myriad stromal cell types and impressive biochemical and structural complexity. This review dissects the cellular and physical components of the intestine and discusses bioengineering approaches for replicating them in vitro to create advanced models of the intestine for disease modelling and regenerative therapies.

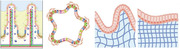

## 1 Introduction

**Figure 1:**
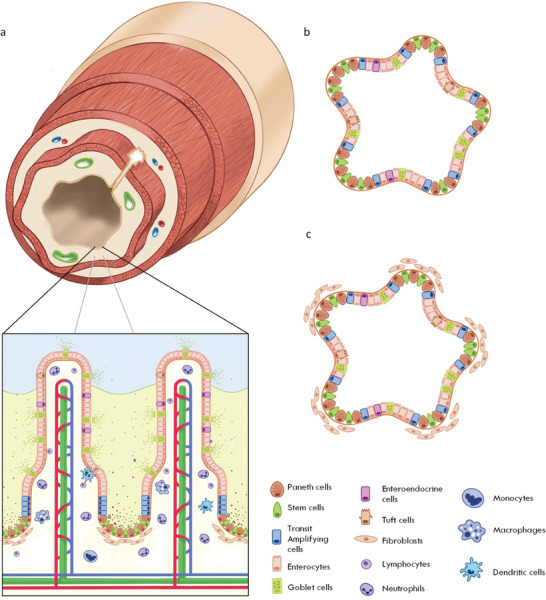
The intestinal epithelium and intestinal organoids. a) Cross section of the small intestine with a zoomed schematic of the intestinal epithelium. b) Adult stem cell‐derived intestinal organoid containing differentiated epithelial cells with both secretory and absorptive phenotypes. c) Embryonic or pluripotent stem cell‐derived intestinal organoid containing differentiated epithelial cells and surrounding mesenchymal cells.

The intestine is central to human health and consists of the small intestine, which absorbs nutrients from digested food, and the large intestine, which supports reabsorption of water and ions. Together the intestine constitutes a selective barrier against the gut's contents and houses the largest portion of the microbiome in mammals. The intestine maintains this barrier by means of a single layer of secretory and absorptive intestinal epithelial cells (IECs), which are supported by an underlying stromal tissue containing matrix‐secreting mesenchymal cells, neurons, smooth muscle, vasculature, and a plethora of immune cell types (**Figure** **1**a).

Defective intestinal barrier function contributes to diverse pathologies including autoimmune diseases, neuropsychiatric disorders,^[^ ^1^ ^]^ inflammatory bowel disease (IBD),^[^ ^2^, ^3^ ^]^ cystic fibrosis,^[^ ^4^ ^]^ and colorectal cancer (CRC).^[^ ^5^ ^]^ Therefore, research models of the intestine are critical for understanding these diseases and informing drug development. However, animal models are limited by in vivo complexities and phylogenetic differences, and standard 2D cell culture models cannot re‐create the complexity and diversity of the native tissue.

More than a decade ago it was shown that the intestinal epithelium (IE) could be reproduced in vitro as intestinal organoids (IOs) (Figure 1b,c). IOs can be cultured indefinitely as 3D structures that display native‐like apicobasal polarity, and quickly proved to be accessible tools to study intestinal development. Over time, IOs revealed insights into how genetic variants influence tissue homeostasis, and allowed for the mechanisms tissues harness to respond to damage to be uncovered. Importantly, IOs also offered new opportunities for researchers to model native tissue‐like responses to disease. In short, IOs have overcome many drawbacks of traditional 2D cell culture and animal models, and have proven to be instrumental tools in intestinal research. However, most IOs consist of a single layer of epithelial cells, while the native IE is supported by numerous mesenchymal and immune cell types, which arrange themselves within a biochemically complex and structurally intricate extracellular matrix (ECM). Hence, researchers are developing new approaches that incorporate this plethora of physiological complexities into in vitro models of the intestine to answer questions that IOs alone cannot address.

Here, we discuss the cell types and matrix components that comprise the intestine and introduce research approaches to re‐create them in vitro. We highlight strategies that rely on the inherent self‐organization capacity of IOs for forming the epithelium, and present these in conjunction with designed approaches from bioengineering that aim to build tissues *de novo*. We discuss how biophysical cues within the native intestinal tissue, and the plethora of biochemical, gaseous, and microbial gradients in the intestine contribute to normal function and can be replicated in vitro. We also highlight how new materials, including hydrogels with time‐dependent and on‐demand modulation of chemical and biological cues, can be used to mimic the physiochemical and mechanical properties of the native ECM. We end by providing a perspective on how further advances in biology might be combined with innovations in bioengineering to create advanced models of the intestine that can rival the accuracy of native tissues for applications in drug screening and personalized medicine.

## 2 Cells and Soluble Signaling within the Native Intestine

The intestine consists of the epithelium that lines the lumen, as well as the underlying lamina propria and deeper submucosal and muscular layers, which together contain multiple mesenchymal cell types with distinct localizations and functions. This includes fibroblasts, myofibroblasts, pericytes, smooth muscle cells (SMCs), enteric neurons, endothelial cells, and immune cells. The intestine also contains a critical cell‐free actor: the mucus. This glycoprotein network lines the luminal tube and acts as a first line of defence against mechanical, biological and chemical assaults.^[^ ^6^, ^7^ ^]^ In addition, the gut houses trillions of microorganisms, which in conjunction with the intestinal mucosal immune compartment, tolerate and even use the microbiota to the advantage of the organ.

### 2.1 The Intestinal Epithelium and Stem Cell Niche

The intestinal epithelium maintains a strict barrier between the luminal contents and the rest of the body while still allowing for nutrient absorption. IECs achieve this by closely regulating the passage of luminal contents across their apical membrane, while simultaneously tightly sealing off intercellular spaces with selectively permeable tight junctions.^[^ ^8^ ^]^ While the colonic epithelium is flat, the absorptive potential of the small intestine is maximized by luminal protrusions called villi. The inhospitable luminal environment demands continuous replacement of IECs. To achieve this, intestinal stem cells (ISCs) that reside within proliferative invaginations called crypts of Lieberkühn provide this source of cells.

The identity of ISCs remained controversial until lineage tracing revealed that the leucine‐rich repeat‐containing G‐protein coupled receptor 5   (Lgr5) was specifically expressed by cycling crypt‐base columnar cells that can generate all epithelial lineages of the small and large intestine.^[^ ^9^ ^]^ In the small intestine, ISCs reside at the base of crypts and fuel the turnover of the epithelium by continuous self‐renewal or generation of daughter cells that rapidly proliferate before terminal differentiation. These daughter cells are either absorptive progenitors that differentiate into enterocytes, or are secretory progenitors that give rise to mucus‐secreting goblet cells, antimicrobial peptide‐secreting Paneth cells, hormone‐secreting enteroendocrine cells, antigen‐binding M cells and cytokine‐secreting tuft cells.^[^ ^10^, ^11^ ^]^ With the exception of Paneth cells, which migrate downwards intercalating between ISCs in the crypt, the other differentiated IECs migrate upward along the crypt‐villus axis eventually reaching the villus tip, where they are shed into the lumen. This process feeds a cellular conveyor belt that replenishes the crypt‐villus axis every 5–7 days in humans and maintains homeostatic barrier function.^[^ ^8^ ^]^

Stem cells within intestinal crypts replenish the epithelium; however, the ISC niche itself plays the more crucial role in this homeostatic process. The ISC niche comprises the physical and cellular microenvironment surrounding ISCs, which provides biochemical and mechanical cues that maintain ISC self‐renewal, proliferation, and organized differentiation. Cells pushed out of the ISC niche are exposed to a signaling environment that promotes quiescence and differentiation, whereas cells that remain within the niche are proliferative and multipotent. Indeed, evidence suggests that the niche is so instructive that if space becomes available, even enterocyte precursors can revert into stem cells in response to niche signals.^[^ ^12^ ^]^

### 2.2 Signaling, Gaseous, and Microbial Gradients Along the Crypt‐Villus Axis

Multiple signaling pathways are arranged as complex gradients along the crypt‐villus axis that are established and maintained by soluble factors released by epithelial and mesenchymal cells. The most important of these include Wnt, Notch, epidermal growth factor (EGF), Eph/Ephrin, and bone morphogenic protein (BMP) signaling. Moreover, the mucus layer that lines the epithelium enables the continuation of gaseous gradients emanating from the enteric blood supply (most notably oxygen), while also providing a medium for gradients of gaseous and molecular microbial products emanating from the lumen.

#### 2.2.1 Wnt Gradients

Wnt signaling is critical for regulating the self‐renewal, proliferation, and maintenance of ISCs during homeostasis. Wnt signaling is established as a gradient, which increases toward the crypt and is actively suppressed toward the villus. Paneth cells are multifunctional cells that both secrete antimicrobial lysozymes and defensins into the crypt lumen, and are a major source of Wnts.^[^ ^13^, ^14^ ^]^ Wnt ligands have a limited diffusive range, thus Paneth cells are intercalated between ISCs so that they can provide Wnt ligands directly to their neighboring ISCs. In addition to Paneth cells, Wnt ligands can also be provided to the niche by pericryptal mesenchymal cells, including telocytes, as well as Gli1^+^ and CD34^+^ cells.^[^ ^15^, ^16^, ^17^ ^]^ Similarly, pericryptal stromal cells also provide Wnt agonists R‐spondins that control ISC self‐renewal.^[^ ^18^, ^19^, ^20^ ^]^ Together, this cellular control maintains a Wnt signaling gradient with high activation at the crypt‐base, which decreases toward the villus tip.

#### 2.2.2 Notch Gradients

Notch signaling also exists as a gradient, which increases toward the crypt, and plays an important role in controlling cell fate in the ISC niche. Combined high levels of both Wnt and Notch signaling maintain ISC identity. However, upon displacement from the crypt, progenitors make fate decisions based on Notch activation.^[^ ^21^ ^]^ Low Notch activation leads to the production of Notch delta‐like ligands (Dll1 and Dll4), which activate Notch in neighboring cells, favoring secretory lineage commitment. Alternatively, high Notch activity results in absorptive cell differentiation. Paneth cells are key drivers of Notch signaling as they provide Dll1 and Dll4, which bias absorptive lineage commitment.^[^ ^22^ ^]^ Thus, Paneth cell localization maintains the correct proportions of absorptive and secretory cell types in the epithelium. Importantly, while low Notch signaling biases toward a secretory lineage, the Wnt signaling context dictates which secretory lineage cells adopt. Thus, positioning is crucial so that Paneth cells, which depend on high Wnt signaling, arise near the crypt, whereas goblet cells are more abundant in the villus.^[^ ^23^ ^]^

#### 2.2.3 EGF Gradients

EGF is another soluble factor that establishes a signaling gradient with the highest activity toward the crypt‐base. EGF belongs to a family of eleven structurally related ligands that encourage the formation of homo‐ and hetero‐dimers between four distinct ErbB receptors. In turn, these dimers stimulate intracellular signaling pathways known to be involved in cell death, proliferation, and cell fate decisions.^[^ ^24^ ^]^ Because of this complexity, although it is recognized that EGF plays a role in maintaining the proliferative status of ISCs, the roles played by other EGF family ligands and receptors in the intestine are not well understood. Indeed, inhibition of EGFR or withdrawal of EGF from murine IOs dramatically reduces proliferation and induces quiescence and an enteroendocrine signature in ISCs.^[^ ^25^ ^]^ However, knockout of EGF in mice and intestine‐specific loss of individual ErbB receptors both produce only minimal phenotypic effects.^[^ ^24^ ^]^ This is in contrast to loss of EGF‐family member neuregulin 1, which reduces the proliferation of ISCs.^[^ ^26^ ^]^ These processes are made even more complicated by endogenous production of EGF‐family ligands, and by the fact that the role of individual EGF family members also appears to be species specific. For example, epiregulin is highly expressed in developing human intestinal crypts and promotes the formation of crypt‐like structures in human biopsy‐derived IOs.^[^ ^27^ ^]^

#### 2.2.4 BMP Gradients

BMP signaling gradients also exist in the intestine and play roles in regulating the proportion of terminally differentiated IEC subtypes and in compartmentalizing the ISC niche to the crypt. Loss of Bmpr1a affects the differentiation of Paneth cells, resulting in lower expression of lysozyme and other Paneth cell markers.^[^ ^28^ ^]^ ISCs are also particularly sensitive to BMP signaling, which serves as a critical checkpoint to prevent hyperproliferation. Through Smad‐mediated repression of stem cell signature genes, BMP signaling ensures appropriate ISC self‐renewal during homeostasis and regeneration.^[^ ^29^ ^]^ In contrast to Wnt, Notch, and EGF, BMP signaling is held in a gradient with highest activity toward the villus tip and lowest at the crypt‐base. BMP4 is expressed throughout the lamina propria, whereas its cognate receptor Bmpr1a is expressed by IECs along the villus and in ISCs.^[^ ^30^ ^]^ This inhibits Wnt target genes, promoting quiescence and permitting differentiation of IECs exiting the transit amplifying zone.^[^ ^31^ ^]^ Meanwhile, pericryptal intestinal subepithelial myofibroblasts and SMCs counteract BMP signaling in the crypt through paracrine secretion of BMP antagonists, Gremin, Noggin, and Chordin‐like proteins.

#### 2.2.5 Eph/Ephrin Gradients

Eph/Ephrin interactions are contact‐based, cell‐cell interactions best known for providing repulsive cues during developmental processes including axon pathfinding and neural crest cell migration.^[^ ^32^, ^33^ ^]^ In the intestine, EphB2 and EphB3 expression in concentrated in cells populating the crypt and decreases toward the villi. Conversely, EphrinB2 and EphrinB3 expression are highest at the villus tip. This expression profile reflects a functional transition between proliferative and differentiated epithelial cells. Disruption of EphB/EphrinB signaling leads to intermingling of proliferative and differentiated cells and loss of downward migration of Paneth cells.^[^ ^34^, ^35^ ^]^ Thus, differential expression of Eph/EphrinB sets up a gradient that controls cell positioning along the crypt‐villus axis during development and in homeostasis. There is also evidence that EphB signaling promotes proliferation of intestinal progenitor cells by inducing cell‐cycle re‐entry.^[^ ^36^ ^]^

#### 2.2.6 Gaseous Gradients

In addition to signalling pathway gradients, there are also gaseous gradients along the crypt‐villus axis. For example, oxygen diffuses from the submucosa where pressures are 80–100 mmHg at the crypt‐base to the villus tip where they are <10 mmHg. Indeed, the luminal side of the IE is hypoxic under homeostatic conditions, and adapts its energy requirements to a low‐oxygen environment.^[^ ^37^ ^]^ Oxygen availability influences nitric oxide (NO) chemistry, which plays roles in immune modulation, mucus production and epithelial secretions.^[^ ^38^ ^]^ Oxygen levels also influence the type of microbiota in the gut. Indeed, low oxygen in the colonic lumen provides an environment for anaerobic microbes that produce gaseous gradients of methane, H_2_ and H_2_S.^[^ ^39^, ^40^ ^]^ These anaerobic gases are implicated in various diseases,^[^ ^40^ ^]^ and like NO, H_2_S is a gasotransmitter at very low levels with reported prosecretory, smooth muscle relaxant and anti‐inflammatory effects in the intestine.^[^ ^41^ ^]^

#### 2.2.7 Microbial and Microbial‐Derived Products Gradients

The gut houses a complex ecosystem with hundreds of species of commensal bacteria that play roles in nutrient absorption, metabolism, immune regulation, and protection against potential pathogens. Imbalances in the microbial community can contribute to a wide variety of pathologies. The small intestine is more acidic and employs more antimicrobial defense mechanisms than the colon, hence it houses comparatively fewer flora and is dominated by fast‐growing facultative anaerobes. The colon contains the denser and more biodiverse portion of the gut microbiome, and is dominated by species such as *Bacteroides thetaiotaomicron* that can use polysaccharides as a carbon source. Myriad microbes and microbial products exist in gradients throughout the intestine, increasing closer to the lumen. In this environment, activation is continuous, thus soluble factors produced by IECs diffuse out into the lumen and exist in an inverse gradient with microbial products. Moreover, metabolites of commensals also exist in gradients across the IE and are implicated in maintenance of barrier function.

### 2.3 Stromal and Other Cell Contributions to the Intestine

Underlying the epithelium are cell‐ and ECM‐rich layers of tissue containing myriad stromal cells. These include fibroblastic cells, which create and maintain the ECM, vascular and neuronal cells, as well as numerous innate and adaptive immune cell populations.

#### 2.3.1 Fibroblastic, Vascular, and Nervous Tissue Cells

Fibroblasts and myofibroblasts dominate the lamina propria, the tissue that underlies and supports the epithelium, and play important roles in wound healing. These cells are both key sources of ECM components and ECM remodeling factors such as matrix metalloproteinases (MMPs).^[^ ^42^ ^]^ Indeed, fibroblasts create the layered structure in the lamina propria, which is characterized by the presence of specialized conformations of the ECM, and contains morphologically and transcriptionally distinct cell populations that reside in each layer.^[^ ^43^ ^]^ Intestinal subepithelial myofibroblasts (ISEMs) express vimentin and α‐smooth muscle actin in the absence of desmin, and their contractility influences the biochemical and mechanical nature of the mesenchyme. Under the lamina propria and in a layer separating it from the submucosa are SMCs. SMCs are responsible for peristalsis, which moves food contents along the gastrointestinal tract. They also continuously relax and contract to eject microbes and secreted material from the crypts, helping to regulate host‐pathogen interactions. SMCs and ISEMs are also both important sources of trophic factors such as BMP antagonists, which maintain the ISC niche.^[^ ^44^, ^45^ ^]^

Underlying the SMCs and within the submucosa are enteric blood vessels and their associated pericytes. Blood vessels provide nutrients to the tissue and deliver circulating immune cells to the gut, which can directly induce intramucosal immune responses.^[^ ^46^ ^]^ Further, endothelial cells are thought to be important for ISC homeostasis, since endothelial cell survival following radiation‐induced injury is required for ISC survival in mice.^[^ ^47^ ^]^ Intestinal vasculature constitutes an additional barrier isolating the contents of the gut from the rest of the body and is also critical for absorption and transport of vitamins, cholesterol, and gut hormones.^[^ ^48^ ^]^

The enteric nervous system (ENS) consists of two sub‐networks, the myenteric and submucosal plexuses, that together are capable of local autonomous function.^[^ ^49^ ^]^ Many types of enteric neurons are found in the intestine including intrinsic primary afferent neurons, motor neurons, interneurons, and enteric glia cells. Together, enteric neurons innervate submucosal muscle to regulate gut motility and peristalsis. Moreover, secretomotor neurons that innervate the mucosa control its permeability to ions.^[^ ^49^ ^]^ This occurs in conjunction with the efforts of enteric vasodilator neurons that ensure mucosal blood flow is appropriate to balance nutrient needs with fluid exchange between the vasculature, the interstitial fluid, and the gut lumen.^[^ ^50^ ^]^ Enteric glia cells, on the other hand, play a neuroprotective role by regulating intestinal barrier function and secreting proinflammatory cytokines.^[^ ^51^ ^]^

#### 2.3.2 Intestinal Immune Cell Populations

The intestine is rich in immune cells, and mucosal immunity is an expansive and complex field. For the interested reader, these cells are covered in more detail elsewhere.^[^ ^52^ ^]^ Briefly, however, numerous specialized innate and adaptive immune cell populations including mast cells, innate lymphoid cells (ILCs), macrophages, dendritic cells (DCs), T cells, and B cells participate in bidirectional interactions with IECs and commensal microbiota to cultivate a tolerant mucosal immunity which regulates, maintains, and regenerates the mucosal barrier.

A particularly abundant immune cell population in the intestine is macrophages. While macrophages are present across the body, those of the gut lamina propria are replenished specifically from recruited Ly6C^+^ blood monocytes,^[^ ^53^ ^]^ which mature into macrophages in the lamina propria.^[^ ^54^ ^]^ These macrophages produce interleukin‐10 (IL‐10), which promotes macrophage toll‐like receptor (TLR) hyporesponsiveness, drives differentiation of T_reg_ cells, and promotes survival of antigen‐tolerant FoxP3^+^T_reg_ cells.^[^ ^55^ ^]^ During inflammation, normal Ly6C^+^ monocyte maturation is disturbed, and both monocytes and macrophages produce proinflammatory cytokines which stimulate TLR responsiveness and their conversion to proinflammatory effector cells.^[^ ^56^, ^57^, ^58^, ^59^ ^]^

Since the gut hosts foreign material, it is important for gut‐associated lymphoid tissue (GALT) to avoid overactivity. Importantly, lymphoid‐tissue‐resident commensal bacteria including *Alcaligenes* spp. are thought crucial for mucosal tolerance.^[^ ^60^, ^61^, ^62^, ^63^ ^]^ For example, *Alcaligenes* spp. resides inertly within DCs participating in immunosurveillance by stimulating production of IL‐6 and transforming growth factor‐β (TGF‐β), and promoting tolerance by suppressing the activity of Th17 cells.^[^ ^60^, ^61^ ^]^ Moreover, DCs produce IL‐23, which in turn induces IL‐22 production by type 3 ILCs and leads to secretion of antimicrobial peptides by IECs, which limit colonization by other bacterial species.^[^ ^64^ ^]^ Moreover, within follicle‐associated epithelium that form dome‐like structures called Peyer's Patches, specialized M cells sample contents of the lumen and release them to the basal extracellular space by transcytosis.^[^ ^65^ ^]^ M cells possess microfolds on their basal surface that engulf DCs which process these antigens and present peptide‐epitopes to CD4^+^ T cells, inducing adaptive immune responses.^[^ ^66^ ^]^

## 3 The Physical Niche of the Native Intestine

The intestinal ECM provides structural support to the tissue and physicochemical cues to the ISC niche. This physical niche consists of the basement membrane and the underlying lamina propria. The basement membrane primarily consists of laminins, collagen IV, nidogen, tenascin‐C and fibronectin, which are tightly integrated into a structure that anchors the epithelium to the lamina propria. The lamina propria is composed of loosely‐associated laminins, fibrillar collagens, fibronectin and glycosaminoglycans including heparan sulfate proteoglycans (HSPGs) and hyaluronic acid (HA).^[^ ^51^ ^]^ In addition to providing architectural support, the intestinal ECM acts as a reservoir for growth factors and a dynamic medium for cells to respond to both biochemical signals and mechanical forces.

### 3.1 Intestinal ECM Composition

Collagens are an abundant superfamily of proteins and critical structural component of tissues. Collagen types I, III, IV and VI (ColI, ColIII, ColIV and ColVI) are abundant throughout the intestine; however, differential expression by epithelial and mesenchymal sources distinguishes the basement membrane where ColIV and ColVI are found, from the lamina propria where ColI and ColIII predominate.^[^ ^67^, ^68^, ^69^, ^70^, ^71^, ^72^ ^]^ This segregation suggests that ColIV and ColVI may help define the physical ISC niche, which is supported by evidence that IEC‐specific ColVI deletion leads to changes in cell morphology and migratory status that interrupt crypt homeostasis.^[^ ^72^ ^]^ Collagens are also critical for damage repair. For example, in response to injury, ISEMs regulate the deposition of collagens, which reinforces barrier integrity and enables wound healing. After injury resolution, excess ISEMs apoptose and collagen levels return to homeostatic levels.

Laminins are glycoproteins abundant in the basement membrane, and whose subtypes exhibit differential spatial and temporal expression patterns that are species specific. Laminin‐α1 and ‐α2 are enriched in the basement membrane of crypts across the murine intestine, whereas laminin‐α5 is localized to the villus region. Conditional knockout of laminin‐α5 in the subepithelial basement membrane of mice leads to ectopic deposition of colonic laminin‐α1 and ‐α2, and the loss of villus architecture in the small intestine.^[^ ^73^ ^]^ Contrastingly, in humans, laminin‐α1 and laminin‐α5 are localized to the villus and present early in development, while laminin‐α2 expression coincides with the development of crypts.^[^ ^74^, ^75^ ^]^ Thus, laminins play key roles in intestinal physiology that are specific to distinct areas of the tissue.

Fibronectin is another key component of intestinal ECM and is secreted by both fibroblasts and IECs. It contains multiple binding sites for glycosaminoglycans, collagen and RGD‐binding integrins.^[^ ^76^ ^]^ In the mouse intestine, fibronectin is upregulated in response to inflammation and can induce nuclear factor‐κβ expression, which is thought to assist with wound healing by activating ISEMs.^[^ ^76^ ^]^

The major glycosaminoglycans in the intestinal ECM are HSPGs and HA. HSPGs are linear polysaccharides primarily localized in the BM, of which perlecan is predominant.^[^ ^77^ ^]^ HSPGs bind growth factors such as Wnts, Hedgehog ligands, TGF‐β and fibroblast growth factor (FGF).^[^ ^51^ ^]^ Indeed, heparan sulfate increases the binding affinity of IECs to Wnt ligands in mice, assisting regeneration following irradiation.^[^ ^78^ ^]^ HA is a non‐branching polysaccharide composed of disaccharide repeats of d‐glucuronic acid and N‐acetyl‐d‐glucosamine. HA is abundant in many tissues, often playing roles in tissue hydration. However, in the intestine, HA also plays key roles in regulating immune responses. HA can be cleaved into fragments which are recognized by toll‐like receptors 2 and 4 (TLR2/4), which induce the production of proinflammatory cytokines and chemokines.^[^ ^79^ ^]^ Pericryptal macrophages expressing TLR4 can sense HA fragments, triggering signaling which ultimately drives ISC proliferation and crypt fission.^[^ ^80^ ^]^ Moreover, HA and its fragments have been linked to leukocyte infiltration and activated myofibroblast‐mediated fibrosis.

### 3.2 Enzymatic Regulators of the Intestinal ECM

The intestinal ECM constantly undergoes remodeling, changing its biochemical and mechanical properties during both homeostasis and in disease. Indeed, balancing ECM deposition against degradation must remain responsive and proportional to challenges such as injury and inflammation. Numerous enzymes directly and indirectly influence the breakdown, deposition, and structure of the intestinal ECM.

#### 3.2.1 Physiological ECM Remodeling

MMPs are a family of proteolytic enzymes with 23 members in humans that, among other functions, degrade ECM components.^[^ ^81^ ^]^ Their destructive nature means MMP expression and activity must be tightly controlled. This is accomplished through a combination of post‐translational modifications, miRNAs,^[^ ^82^ ^]^ and enzymatically by broad‐spectrum tissue inhibitors of metalloproteinases (TIMPs).^[^ ^81^ ^]^ Other degradative proteases, such as anchored metalloprotease‐disintegrin proteins (ADAMs) also regulate the intestinal ECM. ECM remodeling can also be mediated by crosslinking. For example, tissue transglutaminase catalyses the formation of γ‐glutamyl‐ɛ‐lysine isopeptide bonds between and across polypeptide chains,^[^ ^83^ ^]^ while lysyl oxidase and lysyl oxidase‐like enzymes crosslink collagen fibrils and elastin by deaminating lysine and hydroxylysine residues.^[^ ^84^ ^]^ Together this creates a dynamic ECM which is critical for intestinal homoeostasis and regeneration.

In response to injury, expression of MMPs and their inhibitors are regulated in a highly controlled manner. For example, expression of TIMP‐1 and TIMP‐2 are initially increased, suggesting that countering the action of MMPs is important for wound healing.^[^ ^85^ ^]^ Thus, TIMP expression may favor initial collagen deposition by activated ISEMs. Collagen deposition reinforces barrier integrity during the acute response to damage and allows wound‐associated epithelial cells to populate the wound bed. ISCs from neighboring crypts then migrate to replace these cells and stimulate regeneration, and mesenchymal cells localize to the stroma underlying the wound bed where they limit expansion of the stem cells.^[^ ^86^ ^]^ After the acute restitution phase, MMP2 and MMP9 expression are then elevated until repair is complete.^[^ ^87^ ^]^ Thus, a combination of ECM remodeling and orderly differentiation of IECs enable crypt morphogenesis and completion of the repair process.

#### 3.2.2 Pathological ECM Remodeling

Dysregulation of ECM remodeling is a common feature of many intestinal diseases. Both Crohn's disease (CD) and ulcerative colitis are characterized by aberrant ECM remodeling. CD is often associated with uncontrolled ECM deposition, which can progress to life threatening strictures, whereas ulcerative colitis can lead to lesions caused by breakdown of the colorectal BM. Accordingly, these forms of IBD may be distinguished by differential expression of ECM remodeling enzymes. For example, MMP7 is elevated in colorectal tissue of CD patients,^[^ ^88^ ^]^ and increased expression of MMP2 and MMP9 have been noted in patients with CD.^[^ ^89^ ^]^ Indeed, MMP9 levels become so elevated in these patients that MMP9 is often considered as a biomarker of the disease.^[^ ^90^, ^91^ ^]^

Atypical remodeling of the ECM is also known to play roles in inflammation and CRC progression.^[^ ^92^, ^93^ ^]^ ADAM17 and ADAM15 expression is increased in IECs of inflamed tissue, whereas ADAM10 is upregulated in the late phases of CRC.^[^ ^94^, ^95^, ^96^ ^]^ ADAM17 is also critical for processing tumor necrosis factor‐α, which influences myofibroblast activation and thereby collagen deposition.^[^ ^97^, ^98^ ^]^ Moreover, as in IBD, both MMP2 and MMP9 are upregulated in CRC.^[^ ^89^, ^99^ ^]^ This upregulation of catabolic enzymes in CRC also correlates with changing ECM composition, as collagen IV is replaced by ColI,^[^ ^93^, ^100^ ^]^ which becomes increasingly crosslinked as lysyl oxidase expression increases as CRC progresses.^[^ ^101^ ^]^

In addition to physically altering the matrix, dysregulation of ECM remodeling also impacts the bioavailability of growth factors. Intestinal ECM components, including HSPGs, bind growth factors and act as a ligand reservoir. Different FGF isoforms have differential affinities for HSPG.^[^ ^102^ ^]^ Indeed, FGF signaling promotes survival and proliferation, and is suggested to maintain stemness in CRC.^[^ ^103^ ^]^ Similarly, various isoforms of vascular endothelial growth factor can bind collagen such that MMP9‐mediated remodeling can prompt normally quiescent vasculature to undergo angiogenesis during cancer.^[^ ^104^, ^105^ ^]^ Together, these findings suggest a pernicious link between inflammation, ECM remodeling, and the development/progression of IBD and CRC. In this context, altered ECM composition and mechanics may drive the development of a fibrotic microenvironment that promotes tumorigenesis, and might explain why IBD patients are at higher risk of developing CRC.

### 3.3 Mechanical Cues within the Intestine

**Figure 2:**
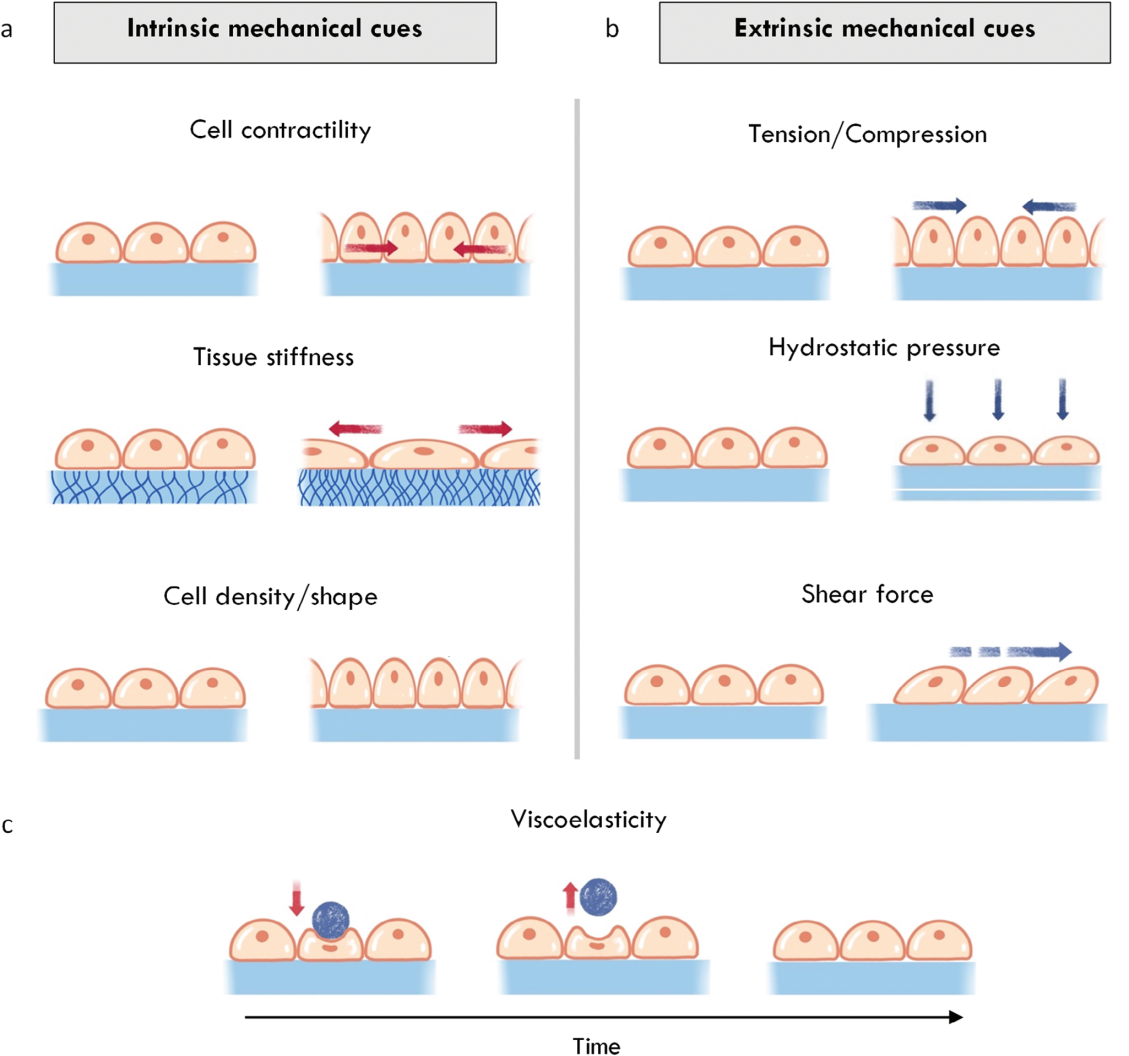
Mechanical cues experienced by intestinal cells. a) Intrinsic mechanical cues. Top: Tensile forces generated by actomyosin contraction apply tension on neighboring cells and the extracellular matrix via focal adhesions. Middle; Stiffness describes the degree to which a material resists an applied force. Bottom: Cell density and shape provide mechanical cues to cells as a result of cell crowding or ECM confirmations. b) Extrinsic mechanical cues. Top: Applied tensile and compressive forces can directly impact cells, for example, by luminal contents applying normal forces to the gut wall as they pass. Middle; Hydrostatic pressures result from fluid itself pushing on a cell. Bottom: Shear forces act parallel to the gut wall and may cause lateral deformation of cells, for example, as luminal contents pass. c) Intestinal tissues are viscoelastic and thus possess properties of both elastic solids and viscous fluids.

The mammalian intestine experiences a range of physical forces that are critical for proper organ function. The intestine is subject to both intrinsic mechanical cues, for example, tissue stiffness (**Figure** **2**a), and responds to extrinsic forces generated by shear flow, compression and hydrostatic pressure^[^ ^106^ ^]^ (Figure 2b). Physical cues act both at the tissue level, impacting organ conformation and function, and at smaller scales whereby mechanical forces are converted into intracellular signals that affect diverse cellular processes including proliferation, differentiation, and migration. The field of intestinal mechanobiology is diverse and excellently reviewed elsewhere.^[^ ^102^ ^]^ Here, we briefly cover mechanical cues within the intestine that are currently being exploited using bioengineering strategies, as discussed in Sections 4 and 5.

#### 3.3.1 Mechanical Properties of Intestinal Tissue

The intestine is a layered composite of concentric tubes: the mucosa, submucosa, tunica muscularis and serosa. The architecture and physical properties of these layers including their stiffness and the tension generated by resident cells, establish the mechanical context of the resting intestine. Stiffness describes a material's ability to resist an applied force. Elastic modulus (*E*) is a size‐independent measure of stiffness and is an intrinsic property of a material. The *E* of a tissue is influenced by both the tissue's resident cells as well as the *E* of the ECM proteins, their density, and their spatial organization within tissue. Healthy colonic tissue has been reported to have an *E* ranging from 0.7 to 0.9 kPa.^[^ ^96^, ^107^, ^108^ ^]^ This contrasts with colonic carcinoma tissue, which has an *E* between 2.4 and 7.5 kPa. Similarly, inflamed regions of intestine appear to be as much as twice as stiff as uninflamed.^[^ ^96^ ^]^ Stiffening has also been observed in the intestines of CD patients, where strictures have been reported with *E* as high as 16.7 kPa.^[^ ^109^ ^]^

However, stiffness alone does not provide a complete picture of the mechanical properties of intestinal tissue. As with other soft tissues, the intestine is viscoelastic, meaning that the tissue displays time‐dependent mechanical properties (Figure 2c). For example, in response to an applied strain, the tissue will undergo stress relaxation, or reduce its resistance to the deformation over time. Such viscoelastic responses result from the highly hydrated and complex fibrous structure of the intestine.^[^ ^110^, ^111^ ^]^ Thus, compressive forces arising from distension of the lumen, for example, as food passes, are likely dissipated between the mucosa and tunica muscularis by stress relaxation.^[^ ^112^ ^]^

**Figure 3:**
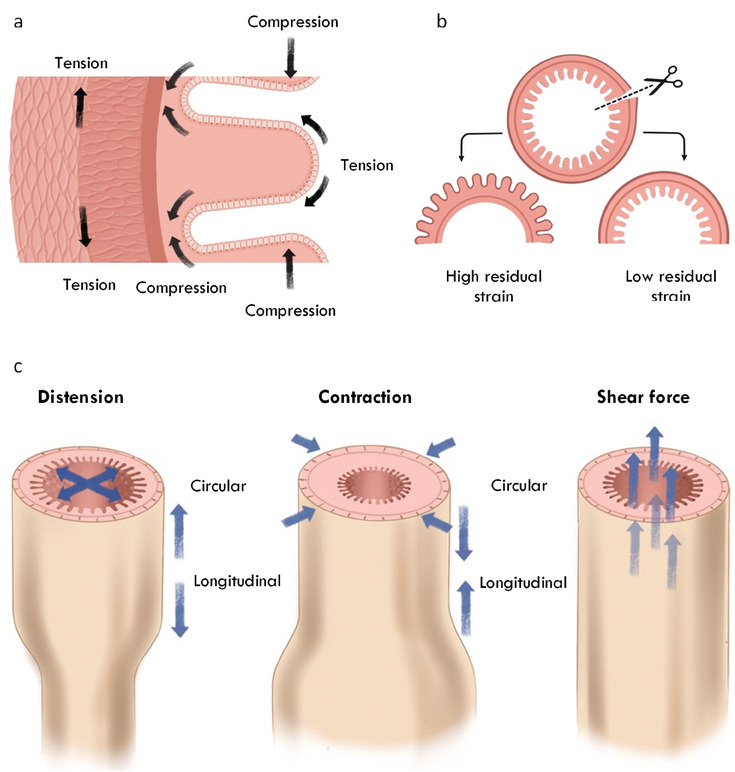
Mechanical forces influencing the static and dynamic intestine. a) Longitudinal cross section portraying the layered organization of the gut wall. The cells of the epithelium experience a balance of tensile and compressive forces. The mucosal and submucosal tissues exist under constant compression both axially from luminal contents and laterally from nearby crypts. Layers farther from the lumen are under constant tension. b) Sections of the intestine can be cut to investigate the residual stress. The opening angle will indicate the balance between compression and tension between layers. c) The dynamic intestine is subject multiple macro‐mechanical processes during peristaltic and inter‐peristaltic periods. Left; Distension of the gut by digested food or microbial metabolism applies outward radial and longitudinal forces. Middle; Contraction of the muscle layers leads to inward radial and longitudinal forces. Right: Shear forces due to the passage of luminal contents act unidirectionally. Adapted with permission.^[^ ^283^ ^]^ 2022, Springer Nature.

The structure of the intestine is designed to accommodate physical forces (**Figure** **3**a). For example, scanning electron microscopy images of rat small intestine show that submucosal collagen fibers are arranged in two threads counter woven at an angle: one clockwise and the other counter‐clockwise, an arrangement suitable for countering forces experienced by the tissue as during digestion.^[^ ^103^ ^]^ Moreover, at rest, intestinal smooth muscle is primed to respond to physical demands by existing in a state of partial contraction.^[^ ^107^ ^]^ This differential tension across the intestinal layers results in the tissue maintaining a level of residual stress, a stress that exists in the absence of applied force.This can be visualized by cutting a section of intestine, which will then pull open nearly instantaneously (Figure 3b).^[^ ^104^ ^]^

#### 3.3.2 Dynamic Mechanical Cues in the Intestine

The intestine is subject to dynamic forces during both active digestion and inter‐digestive periods. At a tissue level, the intestine experiences tensile and compressive forces when the gut wall distends as the lumen is filled by ingested food and secreted material or gas from microbial metabolism (Figure 3c). Distention acts as an extrinsic force on the tissue, which increases its cross‐sectional area and thins the gut wall. This is balanced at a cell level, as cells generate counteracting tensile or “pulling force” via actomysosin contractility The gut also experiences shear forces which act directionally parallel to the epithelial surface as luminal contents pass through. Peristaltic contraction is the main force driving the unidirectional displacement of luminal contents. Segmental contractions of concentric rings of muscle mash and blend luminal contents with gut secretions to make nutrient absorption easier. These contractions occur across the gut and move luminal contents in all directions.^[^ ^108^ ^]^ However, by acting synchronously with contractions of the longitudinal muscle, luminal contents are advanced along the gut tube.^[^ ^105^ ^]^ This luminal flow impacts different layers and areas of the gut differently. For example, the epithelium experiences shear directly, but deeper layers may only experience compressive and tensile forces. Indeed, cells along the villus experience higher levels of shear force than those in the crypts, which are sheltered.^[^ ^109^ ^]^ Similarly, luminal content within the small intestine are more fluid, and exert relatively more shear forces as they pass. This contrasts with the contents of the colon, which are more solid and thus exert relatively more compressive and tensile forces on the tissue.

#### 3.3.3 Mechanically Driven Processes in the Intestinal Epithelium

##### Vilification

Vilification of the intestine takes place during embryonic development. In mice, this process relies on prepatterned and local epithelial‐mesenchymal interactions. Although the exact mechanisms driving this process are unclear, the sub‐epithelial mesenchyme aggregates into dense clusters that promote outward epithelial deformation.^[^ ^113^, ^114^ ^]^ This happens in conjunction with basally directed tension, which pulls the epithelium downwards.^[^ ^115^ ^]^ In combination, the epithelium elongates in response to mesenchymal clustering and this causes compression of epithelial cells between clusters.^[^ ^116^ ^]^ As these cells proliferate and round up, compressive forces move them basally leading to apical invagination at the site of future crypts. While the mechanism governing vilification in humans remains unclear, spatial transcriptomics indicates the presence of a mesenchymal subpopulation that arises beneath emerging villi in a similar manner to mice.^[^ ^117^, ^118^ ^]^

##### Crypt Morphogenesis

Like in vilification, cell‐generated mechanical forces play central roles in crypt morphogenesis. During vilification, proliferative cells are pushed basally and restricted to the intervillus epithelium. The intervillus epithelium then invaginates and ISC precursors become enriched in this region. Compartmentalization of crypts is complete with the development of wedge‐shaped hinge cells that dictate the crypt‐villus boundary. Mysosin II‐dependant apical constriction of intervillus epithelium drives crypt invagination, and actomyosin‐driven basal constriction of hinge cells maintains compartmentalization.^[^ ^119^, ^120^, ^121^ ^]^ Such processes have been modelled in mouse IOs (mIOs) cultured as 2D monolayers on soft surfaces, which organize into crypt and villus‐like zones according to differential cellular tensions.^[^ ^119^ ^]^ Cells within the crypt‐like zone accumulate actomyosin tension and indent the soft substrate in a process reminiscent of crypt formation.^[^ ^119^ ^]^ Together, these observations suggest that both IEC tension and substrate stiffness regulate crypt morphogenesis.

Insight into crypt morphogenesis has also come from investigations into crypt fission. Paneth cells are approximately four times stiffer than ISCs and express significantly higher levels of integrin β4.^[^ ^122^, ^123^ ^]^ Cellular tension applied through the basement membrane by Paneth cells thus regulates crypt wall deformation leading to bifurcation and budding of crypts.^[^ ^123^ ^]^ Indeed, Paneth cell anchoring and tension are thought to mechanically corral ISCs into clusters at the crypt‐base with lower cell tension. As ISCs proliferate these softer areas become prone to buckling in response to compressive forces from nearby crypts and lead to fission events.^[^ ^123^ ^]^ Thus, cell‐mediated deformation of the basement membrane contributes to morphological patterning of the intestinal epithelium in homeostasis. On the other hand, aberrant crypt foci is common in IBD and thought to be the earliest observable change during CRC.^[^ ^124^ ^]^ Activating mutation of the *APC* gene is considered the earliest genetic change in sporadic CRC and alone is sufficient to cause crypt deformity.^[^ ^125^ ^]^ Hence, cellular tension in crypts may also play an important role during intestinal disease.

##### Cell Fate Specification

Soluble factors influence intestinal cell identity; however, evidence suggests mechanical cues also play an important role. Expression of the mechanotransducer Yes‐associated protein (YAP) enhances progenitor proliferation and induces goblet cell differentiation.^[^ ^126^ ^]^ Indeed, mIO‐derived monolayers grown on stiff substrates upregulate expression of YAP and ISC differentiation is skewed toward goblet cells.^[^ ^126^, ^127^ ^]^ Moreover, YAP expression is induced upon injury and dampens the Wnt signaling gradient that normally maintains the stem cell compartment.^[^ ^128^ ^]^ This results in loss of Paneth cells and increased proliferation of progenitor cells. YAP expression has also been shown to be critical for symmetry breaking in mIOs. In this context, stochastic YAP expression is required for a symmetry breaking event, where cells that randomly retain high YAP expression differentiate into Paneth cells, defining the location of a new crypt.^[^ ^129^ ^]^

##### Proliferation, Migration and Shedding

Mechanical cues also influence basic processes such as intestinal cell proliferation, migration, and extrusion. Porcine jejunum cells respond to stretching by proliferating.^[^ ^110^ ^]^ and cyclic strain has been shown to stimulate the proliferation and differentiation of both Caco‐2 cell monolayers and human primary IECs.^[^ ^111^, ^112^ ^]^ Stretch has also been shown to stimulate proliferation of engrafted human IOs.^[^ ^130^ ^]^

Migration is similarly now understood to be a mechanically driven process. Previously, it was thought that proliferative pressure drove IEC migration along the crypt‐villus axis. However, inhibiting proliferation only slows migration of IECs out of crypts and cells still actively migrate up the villus.^[^ ^131^ ^]^ Indeed, traction force mapping of mIO‐derived monolayers has shown that cells are pulled out of the stem cell compartment by traction forces generated in the villus region. Together, these reports suggest that pushing forces from mitotic pressure synergize with pulling forces generated by IECs actively migrating up the villus to drag cells out of the crypt.

During normal IEC turnover, differentiated enterocytes migrating up the villus are eventually extruded into the lumen, where they die due to anoikis, in a process called shedding.^[^ ^132^ ^]^ In the colon, local cell density at the villus tip causes cell compression, which results in extrusion. This is sensed by stretch‐responsive Piezo1 and initiates actomyosin‐driven rearrangement of tight junctions.^[^ ^133^ ^]^ Extrusion is proposed to work like a zipper whereby the extruding cell disassembles E‐cadherin junctions while redistributing tight junctions basolaterally down the membrane.^[^ ^132^ ^]^ Hence, a combination of contractile and compressive forces governs cell shedding.

##### Epithelial Secretions

Coordinating the mechanical processes required for peristalsis and secretion of hormones and mucus is essential for efficient digestion and absorption of nutrients. Enteroendocrine cells are specialized IECs that are thought to be involved in the gut‐brain axis that control these mechanical processes due to their connection with enteric neural synapses and their ability to be electrically excited. Enteroendocrine cells are responsible for sensing and comparing luminal and circulating nutrients to coordinate the secretion of hormones that help control satiety, digestion, and glucose metabolism. Enteroendocrine cells are sparsely populated along the intestine and are categorized into seven subtypes, (K‐cells, L‐cells, delta‐cells, X‐cells, I‐cells S‐cells and N‐cells) based on their hormonal expression.^[^ ^134^ ^]^ Enterochromaffin cells are another subtype thought to sense luminal contents and luminal mechanics. Extrinsic forces sensed by Piezo2‐channels stimulate enterochromaffin secretion of serotonin to promote digestion.^[^ ^135^, ^136^ ^]^ Similarly, goblet cells express Piezo1 and respond to increased hydrostatic pressure in the colon by secreting mucin.^[^ ^137^ ^]^

## 4 Mimicking the Cellular, Biochemical and Structural Components of the Intestine

**Figure 4:**
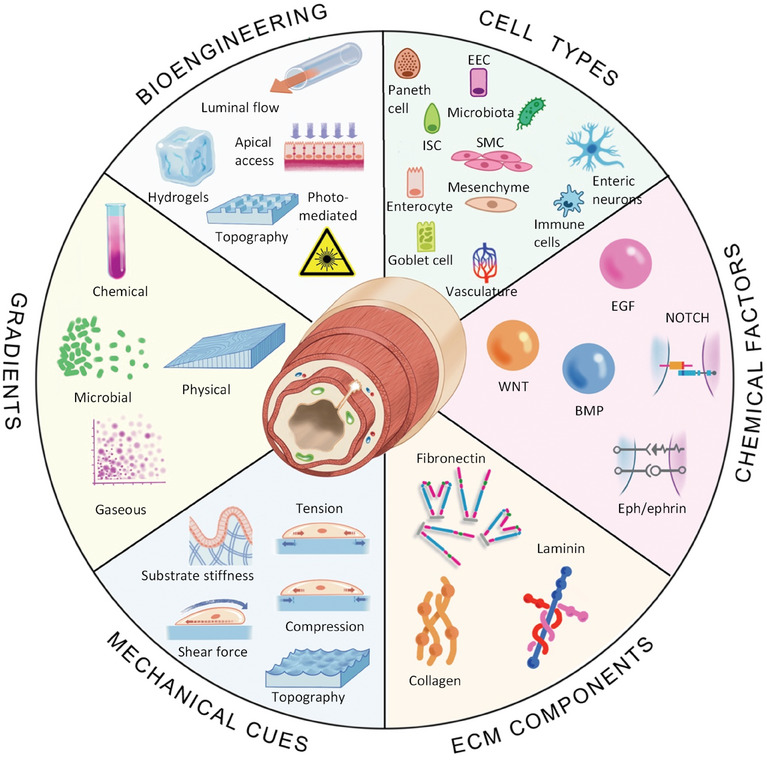
Keys factors for biomimicry of the intestine. The intestine contains diverse cell types that coordinate together to maintain intestinal function. Various chemical factors are critical for maintaining viability and function of intestinal cells. Components of the extracellular matrix provide a physical environment suited to each cell type and their local milieu. The intestine responds to and generates mechanical cues that maintain homeostasis while performing physical tasks. Diverse gradients are established and maintained within the intestine, including along the crypt‐villis axis and across the epithelium, that assist with intestinal homeostasis and defence. Together, interactions between intestinal cells and the chemical, physical, and mechanical features of the intestine is bidirectional and reciprocal, and can be recapitulated using advanced bioengineering technologies including hydrogel chemistries, microfluidics, and light‐based technologies. Enteroendocrine cell (EEC), smooth muscle cell (SMC), intestinal stem cell (ISC).

Building organs in vitro is challenging due to the complexity of native tissue. Indeed, accurately modelling the intestine would necessitate recapitulating cellular heterogeneity and microbial interactions; creating a mechanochemically accurate ECM, biochemical and biophysical gradients, luminal flow, and peristaltic muscle contraction; as well as incorporating all these cues in an accurate 3D architecture alongside vasculature and lymphatics. However, co‐culture models and bioengineering approaches that incrementally combine these cells, features and functionalities with the inherent capacity of IOs for self‐organization are allowing researchers to establish such in vitro models. Drawing from our discussion of the cellular and physical components of the native intestine outlined in Sections 2 and 3, here, we outline strategies to recapitulate the intestine's cellular complexity, and bioengineering efforts to mimic its biochemical composition and structural features (**Figure** **4**).

### 4.1 Recapitulating the Intestinal Epithelium

Prior to 2009, most in vitro models of the IE were tumor derived. Although some cell lines differentiate into monolayers with enterocyte‐like polarity and function, pro‐cancerous selection alters growth, proliferation and differentiation, limiting their use for disease‐specific studies or understanding healthy gut physiology and development.^[^ ^138^ ^]^ Culture of primary IECs more closely recapitulates in vivo cellular heterogeneity; however, these cells cannot be maintained long‐term.^[^ ^139^, ^140^, ^141^ ^]^ Moreover, 2D cell cultures cannot recapitulate the biochemically and mechanically complex 3D environment that ISCs reside within in vivo.

To tackle these obstacles, Sato et al. described methods of recapitulating the ISC niche to support the long‐term culture of IEC.^[^ ^142^, ^143^ ^]^ Mouse small intestinal crypts were embedded within a 3D matrix supplemented with EGF, Noggin, and R‐spondin (ENR medium). This strategy generated cystic structures, now termed IOs, containing ISCs capable of self‐renewal, proliferation, and multilineage differentiation. Moreover, IOs develop architectural complexity and polarity and eventually acquire structural and functional features reminiscent of their parent organ.^[^ ^143^, ^144^ ^]^ This seminal work highlighted the importance of both biochemical and 3D ECM features of the ISC niche and paved the way for the development of IOs from different cell sources.

#### 4.1.1 IO Cell Sources and Applications

IOs can be established from tissue‐resident adult stem cells (ASCs) harvested from healthy or diseased biopsies or cancer cells. They can also be formed from pluripotent stem cells, such as induced pluripotent stem cells (iPSCs). Tissue‐derived cells are harvested by mechanical or enzymatic disruption of tissues, which then self‐assemble into IOs when embedded within 3D matrices and cultured in ENR medium. Prior to seeding in matrix, ISCs are often purified. For example, fluorescence‐based cell sorting of ISCs from transgenic mice expressing Lgr5‐GFP can ensure IOs are formed from the correct progenitor population. Tumor biopsies can be processed similarly; however, sometimes require being grown as xenografts to expand cell numbers sufficiently to generate organoids. Moreover, forming some cancer‐derived organoids can require sorting cancerous cells from normal,^[^ ^145^ ^]^ or using specially formulated media to select for cancer cells. Most research using pluripotent stem cells to form IOs relies on iPSCs. iPSCs are established and maintained as undifferentiated clonal aggregates. To generate human iPSC‐derived IOs (HIOs), iPSCs are first differentiated into FOXA2+/SOX17+ endoderm by supplementation with Activin A. Following this, iPSCs are patterned into CDX2+ mid/hindgut tissue using FGF4 and Wnt3a.^[^ ^146^ ^]^ During the endodermal commitment, mesenchymal cells concomitantly differentiate alongside endoderm, resulting in HIOs containing a hindgut committed epithelial monolayer surrounded by mesenchyme.

##### Modelling Diseases

Various cell populations and structures within the intestine play important roles during infection and are better mimicked by IOs than cell lines. For example, IOs have been used to investigate epithelial‐bacterial interactions by microinjecting bacteria or bacterial toxins into IOs to study interactions with the apical side. Some researchers have also investigated interactions with the basal side by prompting IOs to grow with their apical side facing out, or even dissociated IOs with bacteria or toxins in suspension. These techniques have been used to study IEC interactions with a range of bacterial species,^[^ ^150^, ^151^, ^152^, ^171^, ^173^, ^176^ ^]^ and even viruses.^[^ ^154^, ^155^ ^]^ In these models, IOs recapitulate bacterial/viral invasion, cellular architecture, expression profiles and inflammatory responses that are seen in vivo. Similarly, diseases including colorectal cancer,^[^ ^156^ ^]^ cystic fibrosis,^[^ ^157^ ^]^ and IBD^[^ ^158^ ^]^ have also been modelled using IOs. Targeted CRISPR‐Cas9 gene editing has been used to create mutations that allow for modelling of cancer initiation and progression from untransformed IOs.^[^ ^156^ ^]^ Gene editing has also been used to repair transmembrane receptors in IOs from cystic fibrosis patients.^[^ ^157^ ^]^ Similarly, IOs from patients with CD have been used to test treatments that aim to improve epithelial barrier function.^[^ ^158^ ^]^

##### Biobanking, Personalized Medicine and Drug Screening

The need for personalized medicine, particularly for cancer patients, has prompted the creation of collections of healthy and diseased patient‐derived organoids (PDOs). As patients’ genetic signature can be represented by PDOs and remain stable long‐term, such models enable personalized genetic testing and large‐scale drug screening. Moreover, xenotransplantation can be used to monitor PDO development in more physiologically representative microenvironments. This field remains relatively underdeveloped but already the use of colorectal PDOs has highlighted the potential of such biobanks for screening potentially targetable, but rare genetic variants.^[^ ^159^, ^160^ ^]^

#### 4.1.2 Limitations of IO‐Based Models

Although IOs have proven instrumental in intestinal research, they exhibit heterogeneity within their constituent cells (intra‐organoid heterogeneity) and across different samples (inter‐organoid heterogeneity). Intra‐organoid and inter‐organoid heterogeneity reflect the complexity of biological systems, and thus can be useful for modelling development and regeneration, and for comparing disease states between patients. However, high variability is not appropriate for applications such as drug discovery and precision medicine. Some variability within HIO cultures is thought to be attributable to the presence of the mesenchymal cells, as their abundance is dependent on manual “cleaning” performed during passaging. Development of mesenchymal cells alongside the hindgut‐committed epithelial cells was previously deemed inevitable; however, recent reports describe the generation of HIOs lacking mesenchymal support.^[^ ^161^ ^]^ Using a *CDX2^eGFP^* iPSC knock‐in reporter line enabled researchers to track the emergence of hindgut progenitors and purify them, resulting in mesenchyme‐free HIOs.^[^ ^161^ ^]^ This can help reduce variability compared to traditional HIOs.

Another limitation of HIOs is that they are not representative of the adult intestinal epithelium. Instead, HIO transcriptomic profiles match those of fetal intestinal cells.^[^ ^162^, ^163^ ^]^ Maturation can be enhanced through the addition of IL‐2 and neureregulin; however, cells still remain immature.^[^ ^26^, ^164^ ^]^ Hence, for applications in which adult IECs are required, tissue‐derived IOs may be more suitable than HIOs. Implantation of HIOs into animal models can enhance their maturation,^[^ ^165^, ^166^ ^]^ as it increases cellular complexity;^[^ ^167^ ^]^ however, animal implantation is not suitable in many applications. Other limitations include that for regenerative applications, HIOs are not appropriate, in part due to the potential for generating tumors. Moreover, some IO populations, such as HIO‐associated mesenchymal cells, are poorly characterized, which can lead to difficulty in assessing experimental outcomes.

### 4.2 Incorporating Stromal Populations, Immune Cells, and Microbiota

Intestinal models that aim to include stromal cells often rely on establishing cocultures with epithelial cells. This includes cocultures with pericryptal mesenchymal cells, which provide soluble factors and matrix cues that support the ISC niche. Models have also been established with enteric nervous system, blood vessel, and immune cell populations. Moreover, methods have been developed to study interactions between the hundreds of species of microbiota that live within the gut lumen and intestinal tissues.

#### 4.2.1 Fibroblast and Nervous System Populations

Coculture of IOs with mesenchymal cells or their matrix assists their growth and maturation. For example, murine IOs (mIO) develop larger crypts with improved survival when cultured on matrix secreted by intestinal myofibroblasts.^[^ ^168^ ^]^ Moreover, pericryptal fibroblasts have been shown to support mIO growth.^[^ ^169^ ^]^ On the other hand, HIOs are inherently cocultures since they exist as a heterogenous mix of epithelial and poorly characterized mesenchymal cells.

Other important stromal cells underlying the IE include pericytes, SMCs, endothelial cells, enteric nerves, and myriad immune cells. Incorporating these cells into tissue models of the intestine is less advanced. Nevertheless, there are reports of coculture protocols for the development of IOs containing a functional ENS.^[^ ^170^ ^]^ By combining protocols for the differentiation of human iPSCs to neural crest cells and HIOs, intestinal ENS development can be recapitulated. HIOs with an ENS expressed lower levels of absorptive lineage markers and decreased goblet and Paneth cell marker expression compared to HIOs alone. Moreover, HIOs with an ENS expressed lower levels of TGF‐β, higher levels of EGF, and showed higher rates of proliferation.^[^ ^170^ ^]^ More recently, such protocols have been extended whereby hESCs were differentiated to human colonoids containing an ENS or blood vessels.^[^ ^171^ ^]^

#### 4.2.2 Immune Cell Populations

Arguably more effort has been made in incorporating immune cells into tissue models.^[^ ^172^ ^]^ Resident macrophages are crucial for creating a tolerogenic environment in the intestinal mucosa that maintains and restores homeostasis. Unsurprisingly, there is interest in the mechanisms governing this system and how it is perturbed in diseases like IBD. HIOs basally express monocyte chemoattractant protein‐1, suggesting that the IE itself has chemotactic capacity.^[^ ^173^ ^]^ Supporting this, in cocultures with HIOs, peripheral blood mononuclear cells migrated toward HIOs where they developed transepithelial protrusions.^[^ ^174^ ^]^ Similarly, in a 2D system using human biopsy‐derived IOs (hIO) cultured as monolayers on Transwell membranes, coculture with monocyte‐derived macrophages increased monolayer thickness and improved transepithelial electrical resistance, an indicator of barrier function.^[^ ^175^ ^]^

In addition to macrophages, the intestinal mucosa also houses a plethora of antigen non‐specific lymphocyte populations, including ILCs. Type 1 ILCs (ILC1) are enriched in the intestines of patients with CD.^[^ ^176^ ^]^ By coculturing both mIO with murine ILC1 and HIO with biopsy‐derived ILC1 from patient tissues, intestinal models have been used to show that ILC1‐derived TGF‐β drives proliferation of CD44v6^+^ epithelial cells and mesenchymal matrix remodeling.^[^ ^177^ ^]^ Unravelling this previously undescribed role for ILC1 in the intestine may provide insight into why patients with IBD are at higher risk for cancer and fibrosis. ILC‐organoid cocultures have similarly revealed that ILC‐epithelial interactions are not a one‐way street. In cocultures, both iPSC‐derived intestinal and lung organoids can also signal to ILC progenitors to drive tissue‐specific maturation of the immune cell populations.^[^ ^178^ ^]^

DCs are also important players in the intestine, which researchers have attempted to incorporate into tissue models. DCs interact with the IE, presenting antigens transcytosed by the IE to T cells to mount adaptive immune responses. However, upon activation by foreign antigens, DCs secrete enterotoxic proinflammatory cytokines. Coculture of mIOs derived from *Nfκb2^−/−^* and wild type mice with activated DCs^[^ ^179^ ^]^ highlighted that the IE may modulate NFκB signaling to deal with inflammatory stress caused by DC activation. In another study, mIOs cocultured with DCs were shown to undergo morphological changes and goblet cell depletion with activation of Notch signaling. Direct E‐cadherin‐mediated adhesion of DCs to mIOs led to Notch activation, suggesting a potential target to prevent over‐activation of DCs.^[^ ^180^ ^]^

#### 4.2.3 Microbiota

Including microbiota in cocultures may allow for a clearer understanding of the roles microorganisms play in gut homeostasis and disease.^[^ ^181^ ^]^ To understand how the infant intestine adapts to microbial colonization, HIOs were microinjected with a non‐pathogenic strain of *E coli*.^[^ ^182^ ^]^ The resulting colonized HIOs were more mature, and an interplay between microbial contact and microbe‐associated hypoxia had dramatic effects on the IOs. Indeed, antimicrobial peptide production increased, mucus production matured, barrier integrity was improved and colonized HIOs were more resilient to inflammatory cytokine production.^[^ ^182^ ^]^ These findings improve our understanding of how intestinal development is influenced by microbial colonization.

Many gut microbiota species are anaerobic, which presents challenges for including them in intestinal models. Anaerobes and IOs cannot be cultured within the same media. However, the impact of anaerobes on the epithelium can be studied by microinjecting anaerobes or their bacterial products into the lumen of IOs.^[^ ^152^, ^183^ ^]^ Another approach is to use agar‐epithelial interface cultures that limit oxygen diffusion from the media above to anaerobic bacteria embed within the agar.^[^ ^184^ ^]^ Using a Transwell system separated by an airtight seal and a microporous membrane supporting a Caco‐2 cell monolayer,^[^ ^185^ ^]^ *Faecalibacterium prausnitzii* added to the anaerobic compartment suppressed the inflammatory reaction of Caco‐2 cells, enhancing barrier permeability.^[^ ^185^ ^]^ Similar to Transwells, hemi‐anaerobic coculture systems are also available, which contain a hypoxic apical chamber conditioned with anaerobic gases, which is separated from a normoxic basal chamber. These have been used to grow obligate anaerobes alongside a colonoid monolayer. In this system, *Bacteroides fragilis* was shown to be in competition with the monolayer over glucose. This was in contrast to its interactions with *A. muciniphila*, which were symbiotic and mediated by the metabolism of mucin.^[^ ^186^ ^]^ Despite these advances, static co‐cultures can also lead to the accumulation of undesirable cellular metabolites and microbial overgrowth, which can kill epithelial cells.^[^ ^187^ ^]^ However, by providing luminal flow, viability can be improved, which enables longer studies.^[^ ^188^, ^189^ ^]^

### 4.3 Advances in Recapitulating Biochemical Cues

#### 4.3.1 Biochemical Modulation of IO Phenotype

An exciting approach for modelling the intestine is to enrich IOs for certain cell types. This allows researchers to tease apart the role of specific cell types in health and disease, and enables experiments on cell populations that are otherwise too rare in vivo to study. Such approaches enable experiments that explore the functions of rare IECs, and analyses of cell type‐specific responses to soluble factors, drugs and even coculture.

Currently, supplementation with ENR medium is the minimum culture requirement for mIOs. Taking advantage of knowledge of these soluble cues, researchers have found that addition of Wnt3a to ENR medium in combination with CHIR and valproic acid enriches IOs for ISCs.^[^ ^190^, ^191^ ^]^ Conversely, removing R‐spondin enriches IOs for enterocytes.^[^ ^191^ ^]^ This approach identified transcription factor hepatocyte nuclear factor 4 gamma as a key participant in enterocyte differentiation.^[^ ^192^ ^]^ Similarly, as the Wnt signaling context under which an absence of Notch signaling occurs determines which secretory cell type (Paneth or goblet cells) are generated,^[^ ^23^ ^]^ the addition of the Notch inhibitor DAPT alongside CHIR to ENR leads to development of Paneth cell‐enriched mIOs, whereas adding DAPT and Wnt pathway inhibitor IWP2 enriches for goblet cells.^[^ ^191^ ^]^ This enrichment program was used to compare how the regulatory landscape in mIOs is affected by the differentiation of secretory lineages and revealed key regulators linked with IBD‐associated phenotypes.^[^ ^193^ ^]^

M cells are rare IECs found within follicle‐associated epithelium, which covers GALT, like Peyer's patches. These cells bind antigens trapped in the luminal mucus and deliver them to lymphocytes in GALT for immune surveillance.^[^ ^194^ ^]^ Lineage tracing has shown that receptor activator of nuclear factor kappa‐B ligand (RANKL), which is produced by follicle‐associated epithelium, is crucial for differentiation of M cells.^[^ ^195^ ^]^ Indeed, treatment with exogenous RANKL enriches for M cells, and identified an expression profile that was previously impossible to capture.^[^ ^196^ ^]^ Tuft cells are another rare cell type important for launching type‐2 immune responses to parasitic infection by Helminths. Helminth infection induces IL‐4 and IL‐13 signaling leading to expansion of tuft cells in a positive feedback loop.^[^ ^197^ ^]^ Hence, treatment with exogenous IL‐4 and IL‐13 enriches for tuft cells. Analyses of these cultures were able to identify tuft cell subtypes, characterized by neuronal‐like and immune‐like signatures.^[^ ^196^ ^]^

Supplementation of HIOs with Neurogenin 3 is sufficient to induce differentiation of enteroendocrine cells.^[^ ^198^ ^]^ However, soluble factors to control differentiation were again found using mIOs by combinatorial inhibition of Wnt, Notch and mitogen‐activated protein kinase signaling.^[^ ^199^ ^]^ Indeed, this cocktail enriches for enteroendocrine cells in the crypt, including L‐cells. Alternatively, addition of BMP4 enriches for S‐cells in the villus, and subsequent inhibition of BMP1A leads to enrichment of L‐cells in the villus.^[^ ^200^ ^]^ Despite these advances, enrichment for the full range of enteroendocrine cell subtypes is yet to be achieved.

#### 4.3.2 Recapitulating *I* *n Vivo*‐Like Gradients Across the Intestine

##### Biochemical Gradients

As noted in Section 2.2, soluble and insoluble gradients compartmentalize and maintain the ISC niche. However, in contrast to the gradients of growth factors presented to cells in vivo, standard culture of IOs bathes them in ENR medium resulting in homogenous exposure. To address this, efforts aim to mimic native chemical gradients in vitro using bioengineering approaches. For example, microfluidics has been used to apply gradients of Wnt3a and R‐spondin across murine colonoids, resulting in polarity, with a proliferative side closest to the source of growth factors and a more differentiated side on the other.^[^ ^201^ ^]^ Similarly, human colonic cells have been seeded within microwells formed across a collagen hydrogel placed on a Transwell insert. This enabled the authors to mimic in vivo apicobasal growth factor gradients by placing growth factors in either the upper or lower reservoir.^[^ ^202^ ^]^ More recently, photolithography was used to fabricate villus‐like microstructures consisting of a ColI‐functionalized synthetic hydrogel mounted on Transwell inserts.^[^ ^203^ ^]^ mIO were seeded as monolayers and different culture conditions, including intestinal subepithelial myofibroblast conditioned media, were applied. Here, ISCs and proliferative cells localized to the bases, closer to the source of the growth factors, while differentiated cells localized to the tips.^[^ ^203^, ^204^ ^]^ However, although promising many of these approaches only maintain a gradient in the short‐term as diffusion, even in hydrogels, nullifies gradients over time. Chemically tethering growth factors or providing a constant growth factor source may be more effective strategies to maintain gradients in longer‐term cultures.

##### Gaseous Gradients

Oxygen gradients have been generated in models of the intestine using microfluidics by applying oxygenated media through one microchannel and deoxygenated media in the opposing direction through another channel. This approach has allowed the coculture of a Caco‐2 monolayer on the side fed by oxygenated media and an obligate anaerobe on a membrane above supplied with deoxygenated media.^[^ ^205^ ^]^ In another report, porous silk‐based scaffolds with hollow channels were imbued with human intestinal myofibroblasts and lined by Caco‐2 cells. Here, the cells own respiration was used to generate an oxygen gradient.^[^ ^206^ ^]^

##### Microbial Gradients

Microbial products exist in the intestine as luminal‐submucosal gradients. Building on their previous work recapitulating growth factor graidents,^[^ ^202^ ^]^ Wang *et al*. designed Transwell systems to mimic gradients of butyrate, a product of bacterial fermentation, which diminished stem cell proliferation and promoted their differentiation to absorptive coloncytes.^[^ ^207^ ^]^ Other models have similarly generated gradients across Transwell inserts by co‐culturing hIO monolayers with *E. coli* within the apical chamber and macrophages in the basal compartment. Here, macrophage‐epithelial communication resulted in improved epithelial barrier function and cytokine production, and macrophages were observed sampling the bacterial product gradient across the epithelial interface.^[^ ^208^ ^]^

##### Mechanical Gradients

Although tissue stiffness impacts intestinal function, it is unclear whether stiffness gradients are present across the IE. That is, while differences in collagen content in the colonic submucosa versus mucosa (80% and 30%, respectively)^[^ ^209^ ^]^ suggest that gradients do exist, it is unclear whether such compositional changes result in mechanical or ECM‐ligand gradients in the tissue. To recapitulate 2D stiffness gradients, polyacrylamide hydrogels are often employed as their surface can be coated with ECM proteins and their stiffness modulated over a wide range. One strategy to achieve stiffness gradients is by fabricating a sloping hard substrate on which the polyacrylamide is cast, allowing a gradient of the underlying stiff substrate to be sensed by cells.^[^ ^210^, ^211^ ^]^ Alternatively, inclusion of a photoinitiator can control cross‐linking and thus, stiffness using time‐controlled exposure to UV light. This was recently applied to study collective migration of epithelial cells by durotaxis.^[^ ^212^ ^]^ For a more detailed account of bioengineering approaches for recapitulating mechanical gradients, readers are directed to previous reviews.^[^ ^213^, ^214^ ^]^

## 5 Mimicking the Physical Niche

As described in Section 3, the ECM is an active participant rather than a passive bystander in the intestine during homeostasis and disease. The development of IOs that mimic the epithelium has been mirrored by advances in in vitro systems that more closely recapitulate the physical structure of the intestine. Many approaches rely on using hydrogels, water swollen polymer networks that can encapsulate live cells and mimic many aspects of the native ECM.^[^ ^215^, ^216^ ^]^

### 5.1 3D Matrices to Support Intestinal Cell Cultures

#### 5.1.1 Natural Biopolymers

The most widely used matrix for 3D cell cultures, including IOs, is decellularized ECM (dECM) derived from mouse Engelbrecht–Holm–Swarm tumors, known by commercial names including Matrigel, Geltrex, and Cultrex.^[^ ^217^, ^218^, ^219^ ^]^ Following the discovery that murine ISCs embedded in Matrigel spontaneously self‐organize into mIOs,^[^ ^143^ ^]^ Matrigel has become the industry and academic gold standard. Matrigel is primarily composed of four major ECM proteins: laminin (≈60%) with laminin‐α1 being predominant, ColIV (≈30%), entactin (≈8%), and perlecan (≈2%–3%).^[^ ^220^ ^]^ While ColIV is a key component of intestinal basement membrane, it is Laminin‐α1, which contains binding sites for stem, epithelial, and endothelial cells, that may be responsible for Matrigel's broad support for different organoids, including: kidney,^[^ ^221^ ^]^ brain,^[^ ^222^ ^]^ lung,^[^ ^223^ ^]^ prostate,^[^ ^224^ ^]^ and liver.^[^ ^225^ ^]^

The versatility of Matrigel and its ease of use has made it popular among researchers; however, Matrigel has limitations that require careful consideration. Matrigel's animal origin makes it inappropriate for translational studies, and experiments that could be confounded by immunogenicity. Lactate dehydrogenase elevating virus, for example, infects host murine macrophages and has been found within Matrigel.^[^ ^226^ ^]^ The virus activates TLR7,^[^ ^227^ ^]^ and although TLR7 expression is almost undetectable in murine IECs,^[^ ^228^ ^]^ this underscores limitations for cultures with TLR7‐expressing cells and raises questions about other possible xenobiotic contaminants. Moreover, decellularization is an imperfect process that alongside structural ECM components, leaves behind growth factors, cytokines, and enzymes that may compromise experiments.^[^ ^220^, ^229^, ^230^ ^]^ Matrigel components are enzymatically degradable, thus it is critical for proteases to be excluded, yet MMP7 has been found in Matrigel.^[^ ^231^ ^]^ This is supported by reports that Matrigel films reduce in thickness overtime.^[^ ^232^ ^]^ Most importantly, ECM composition and cellular secretions vary between different animal tumors, thus there is considerable compositional and mechanical variation between Matrigel batches, which also impacts its gelation kinetics.^[^ ^232^, ^233^ ^]^ Thus, although Matrigel remains a critical tool for supporting IOs and modelling the intestine, its limitations have led researchers to seek more controllable ECMs.

An alternative to recapitulating the intestinal ECM with dECM is to use isolated biopolymers including proteins like ColI, or polysaccharides like alginate or HA, which offer more reproducible properties. Acid‐solubilized ColI fibrils self‐assemble at neutral pH, and form hydrogels that are suitable to both establish and maintain murine and human IOs.^[^ ^44^, ^234^ ^]^ ColI hydrogels have also been used to generate human colonoids, and to model the effect of fiber topology on colorectal tumor organoids.^[^ ^235^, ^236^ ^]^ However, while ColI is a key component of the intestinal ECM, it may not be appropriate for the expansion of ISCs or IO culture, since ColI dominates the lamina propria, whereas IECs rest upon a basement membrane composed primarily of ColIV.^[^ ^67^, ^68^, ^69^, ^70^, ^71^, ^72^ ^]^ Moreover, total ColI mRNA expression is higher in inflamed than uninflamed intestinal tissue from IBD patients,^[^ ^100^ ^]^ and ColI increases with CRC stage, while ColIV levels decrease.^[^ ^93^ ^]^ Thus, although ColI can support IO survival and growth, it does not reflect the physiological complexity of the native tissue and may not be appropriate for mimicking the healthy intestinal ECM.

#### 5.1.2 Synthetic Polymers

Fully defined matrices offer the potential for building highly controllable and reductionist mimics of the intestinal ECM. Synthetic polymers are biologically inert and hence act as a “blank canvas” for researchers to chemically “paint” bioactivity. They can also crosslink in the presence of cells using light‐induced or physical or chemical crosslinking methods.^[^ ^216^, ^237^ ^]^ Synthetic hydrogels have the potential to better support mechanistic experiments, as they allow for orthogonal control of mechanical and biochemical cues and reduce the variability inherent in biological components. There have been notable successes along these lines with IOs; however, we have only begun to scratch the surface of hydrogel chemistries that have the potential to recapitulate the intestinal matrix. Readers are referred to alternative reviews for more detailed discussion of hydrogel chemistries.^[^ ^215^, ^218^, ^238^, ^239^ ^]^

Common synthetic materials used to form hydrogels include polyethylene glycol (PEG), self‐assembling peptides, poly(N‐isopropylacrylamide), and poly(vinyl alcohol).^[^ ^218^, ^240^, ^241^, ^242^ ^]^ PEG hydrogels are suitable for IO culture because they are amenable to functionalization. Chemical conjugation of peptides allows these hydrogels to offer RGD‐ and other integrin‐binding motifs, and to incorporate MMP‐cleavable crosslinks.^[^ ^243^ ^]^ PEG makes an effective backbone for hydrogels due to its hydrophilicity and structurally strong yet flexible chain.^[^ ^243^ ^]^ Moreover, PEGs are commercially available with different molecular weights and chemical functionalities, and allow for biocompatible gelation conditions. Thus, it is possible to design PEG networks with variable mesh size and stiffness, and incorporate bioactive sites for cell adhesion, remodeling, and growth factor binding. Moreover, polymerization chemistries such as photoinitiated crosslinking or degradation introduces the possibility for spatiotemporal control over hydrogel structure and mechanics.

### 5.2 Mimicking Physicochemical and Mechanical Cues of the Native Intestinal Matrix

**Figure 5:**
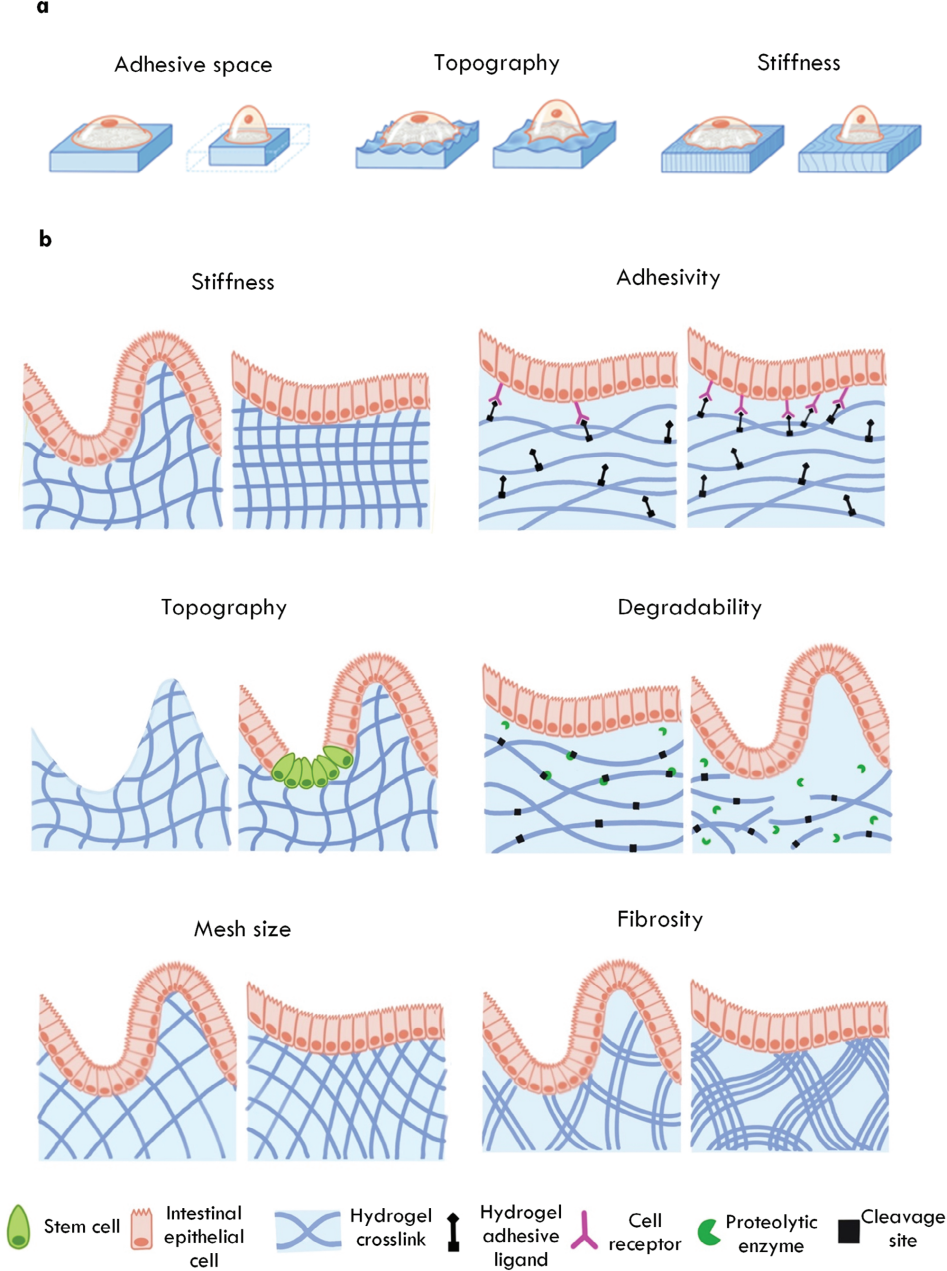
The effects of extracellular matrix cues on intestinal cells. a) Left; Cells with excess space form more focal adhesions, with reinforced cytoskeletal tension whereas cells with limited adhesive space form fewer focal adhesions and adopt a more rounded shape with less cytoskeletal tension. Middle; The more textured the ECM topography, the more focal adhesions cells form. Thus, cells spread on highly textured ECM while fewer topographical features promote rounded cells. Right: A highly crosslinked ECM with a higher stiffness prompts cells to adopt spread morphologies, while softer ECM promotes rounded cells with fewer focal adhesions and lower levels of cytoskeletal tension. b) Top left; Matrices created with lower stiffnesses enable intestinal monolayers to develop crypt/villus‐like morphologies, whereas at higher stiffnesses this morphology cannot develop. Top right: More adhesive motifs may improve intestinal monolayer viability. Middle left: Culture of IOs on matrices fabricated with crypt/villus‐like topographical features promote physiological cellular heterogeneity and localization of progenitor cells. Middle right: Matrices with degradability enable the development of crypt/villus‐like morphology. Bottom left; Matrix features, like hydrogel mesh size, can influence the diffusion of molecules of biologically relevant size. Mesh size can be modulated by altering polymer concentration, molecular weight, and crosslinking density. Bottom right: Matrices can be formed with fibrous structures, mimicking the native ECM. Pathological conditions such as fibrosis may be mimicked this way by altering the size and number of fibers.

Tissue models that incorporate intestine‐like ECM may allow for a better understanding of how matrix cues influence the IE, particularly in modelling fibrotic diseases. While Matrigel contains a plethora of ill‐defined matrix proteins, synthetic and other fully defined matrices allow for matrix proteins and matrix‐mimicking peptides to be introduced systematically (**Figure** **5**).

#### 5.2.1 PEG‐Based Hydrogels

Gjorevski and colleagues were the first to report successfully culturing mIOs in PEG hydrogels.^[^ ^244^ ^]^ The authors found that ISC expansion was not supported in hydrogels lacking adhesive motifs. However, biohybrids incorporating perlecan, HA, ColIV, fibronectin and laminin‐α1, or incorporating RGD sequencing‐containing peptides could all support ISC expansion and mIO formation, although at lower rates than in Matrigel. Sustained culture of mouse and human ISC has also been reported in low‐defect Michael addition PEG‐based hydrogels containing RGD peptides.^[^ ^245^ ^]^ HIO have similarly been reported to remain viable and maintain apicobasal polarity within PEG hydrogels crosslinked with MMP‐degradable peptides and incorporating RGD sequence‐containing peptides.^[^ ^177^ ^]^ Thus, matrix cues derived from native proteins or mimicked using integrin‐binding peptide sequences appear to be required for IO culture.

To determine the best isolated biopolymers for hIO and mIO formation efficiency and yield, hydrogels formed from PEG, HA, fibrin, and alginate have all been tested. None could effectively support colony formation as well as Matrigel; however, fibrin hydrogels supplemented with Matrigel were comparable to 100% Matrigel. In trying to narrow down which component of Matrigel was required for organoid culture, it was found that only fibrin/laminin‐α1 hydrogels supported good IO formation efficiency, maturation, and long‐term passage.^[^ ^246^ ^]^ Therefore, laminin‐α1 may be the critical component in Matrigel's success.

Organoids from other tissues have similarly been used to explore the importance of specific ECM cues in supporting the epithelium in tissue models. For example, PEG hydrogels functionalized with integrin‐binding peptides and crosslinked by MMP‐degradable peptides support the culture of a range of epithelial organoids.^[^ ^247^ ^]^ Inspired by integrin expression by native IECs, hydrogels were created incorporating either integrin α5β1‐binding fibronectin‐derived peptides or ColI‐derived peptides recognized by integrin α2β1, which is predominant at the crypt‐base. To harness interactions with endogenously produced ECM, peptides with affinity for fibronectin or basement membrane proteins were also added to locally sequester secreted proteins.^[^ ^247^ ^]^ Here, the inclusion of the ColI‐derived peptide was critical for IO formation,^[^ ^247^ ^]^ highlighting the importance of mimicking the ISC niche to achieve effective tissue growth.

#### 5.2.2 Recombinant Protein Hydrogels

An alternative option to using Matrigel or synthetic hydrogels is to use materials formed from recombinant proteins. mIOs have been successfully cultured within engineered ECM formed from an elastin‐like structural backbone modified with RGD sequence‐containing peptides.^[^ ^248^, ^249^, ^250^ ^]^ Using this engineered elastin‐like protein (ELP) hydrogel, mIO within high stiffness hydrogels had higher MMP activity and inhibition of MMPs decreased organoid formation,^[^ ^251^ ^]^ suggesting that ECM remodelling by mIOs enables them to adapt to stiff microenvironments. These systems have also been modified by mixing hydrazine‐modified ELP with benzaldehyde‐modified HA, resulting in hydrozone‐crosslinked hyaluronan ELP hydrogels, which supported expansion, maturation and passaging of mIOs.^[^ ^180^ ^]^ Organoids may also secrete supportive ECM proteins themselves, negating the need for an engineered matrix. HIOs have been successfully cultured within alginate,^[^ ^252^ ^]^ which lacks sites for mammalian cell adhesion and remodelling. As HIOs consist of both epithelial and mesenchymal cells, they generate their own ECM. And indeed, within alginate hydrogels, iPSCs formed HIOs with maturity comparable to that of Matrigel‐embedded HIOs.^[^ ^252^ ^]^

#### 5.2.3 3D Hydrogel Mechanical Cues

Although sustained culture of IOs is often reported to be dependent on the presence of specific matrix proteins or biomimetic cues, the stiffness of the 3D matrix also plays a role. For example, ISC colony formation efficiency was shown to be optimal in PEG hydrogels with an elastic modulus of 1300 Pa.^[^ ^244^ ^]^ However, in addition to elasticity, 3D matrices’ viscoelastic, or time‐dependent properties, also impact IOs. Tissues will often deform over time in response to an applied load in a process called creep. Similarly, they will resist an applied deformation less over time, or exhibit stress relaxation. Hydrogels can be designed to mimic these viscoelastic properties by relying on ionic bonding or supramolecular interactions, in which crosslinks can switch off and on, lending them time‐dependent properties.

Viscoelastic hydrogels based on the crosslinking of two different multi‐arm PEG macromers: one covalent, through a Michael addition, and one reversible, through a triple hydrogen bonding interaction were used to culture mIOs^[^ ^253^ ^]^. Seeding single ISCs in both non‐degradable viscoelastic hydrogels and non‐degradable fully covalent hydrogels (elastic) confirmed that colony formation efficiency was enhanced at higher elastic moduli^[^ ^244^, ^253^ ^]^ and insensitive to stress relaxation. However, upon switching to differentiation conditions, colonies within covalently cross‐linked hydrogels developed into cystic structures lacking budding architectures that consisted of enterocytes. In contrast, those within the viscoelastic hydrogels developed budding architectures and cellular diversity associated with mature IOs. Similarly, mIOs grown within alginate hydrogels that rely on ionic bonding allow for normal mIO budding and differentiation, whereas non‐degradable elastic hydrogels resulted in mIO with spherical morphologies.^[^ ^254^ ^]^ These findings suggest that a lack of physical confinement rather than degradability per se is key in mIO differentiation.

In vitro maturation of HIOs beyond the fetal developmental stage is problematic. However, a recent report using iPSC‐derived kidney organoids offers some insight into how by modulating physiochemical and mechanical cues of the 3D matrix, this may be improved.^[^ ^255^ ^]^ Kidney organoids were encapsulated within biologically inert oxidized alginate hydrogels of variable stiffness, as well hydrogels with different stress relaxation properties.^[^ ^255^ ^]^ Similarly to reports on IOs,^[^ ^244^ ^]^ kidney organoids developed better in softer hydrogels, and those embedded within the fastest relaxing hydrogels were the most mature.^[^ ^255^ ^]^ This suggests that stress relaxation is an additional mechanical parameter to consider and one which may benefit matrices designed to mimic the intestinal ECM.

#### 5.2.4 Fibrous Hydrogels

Many synthetic hydrogels are created from small molecular building blocks, like PEG molecules, that assemble at the nanoscale. While these materials create a 3D network akin to the native ECM, they often lack the fibrillar architecture ECM proteins afford to native tissues. Cells are known to migrate along and probe tissue mechanical properties at long distances using thick ECM bands. Hydrogels formed from biopolymers like collagen, provide cells with these fibrous environments and demonstrate non‐linear responses to applied stresses; however, their animal origin, undefined composition, and lack of tunability limits their use. An active area of research aims to mimic the fibrillar structure of the physiological ECM in more defined and even fully synthetic hydrogels using approaches that incorporate fibrous structures within a bulk hydrogel. For example, fibrous hydrogels have been created to disentangle how fiber mechanics and biochemical composition affect chondrogenesis of hMSCs.^[^ ^256^ ^]^ Strain‐responsive properties have also been engineered into fibrous hydrogels to take advantage of cells’ tendency to strain and compact fibrous ECM. Indeed, multifiber hydrogels formed by electrospinning fibers with complementary chemical moieties that form covalent links when brought into close proximity by mechanical load have been used to fabricate macroscale structures.^[^ ^257^ ^]^ More recently, MSCs encapsulated within fibrous hydrogels have been shown to locally recruit fibers leading to macroscale hydrogel contraction.^[^ ^258^ ^]^ To date there have been no investigations into the influence of fibrous hydrogels on IOs; however, there is potential that they will help disentangle the importance of matrix mechanics and ECM composition in intestinal homeostasis and fibrostenotic disease.

### 5.3 Recapitulating Matrix Remodeling during Homeostasis and in Disease

As outlined in Section 3.2, ECM remodeling actively maintains and re‐establishes homeostasis following injury, and is perturbed in disease. Moreover, the ECM acts as a reservoir for growth factors, which upon release can alter cell function. 3D intestine‐specific matrices offer the possibility of modelling these processes in vitro. Many Matrigel components can be degraded by cell‐derived proteases, and because of its undefined composition and as the manufacturer adds protease inhibitors, studies aiming to build in vitro models in Matrigel to understand the role of matrix remodeling in intestinal homeostasis and disease are limited. Synthetic matrices offer the possibility of studying matrix remodeling in a controlled setting as the density and specificity of molecules susceptible to degradation can be controlled.

#### 5.3.1 Engineering Degradable Hydrogels

Many biologically derived and synthetic hydrogels have been created that permit remodeling, often by engineering hydrogels to be degradable. Indeed, although elastic hydrogels support mIO colony formation, spheroids do not develop budding morphologies, and while YAP‐induced proliferation is initially enhanced at high stiffness, this is transient and drops in coincidence with colony failure.^[^ ^244^ ^]^ However, in hydrolytically degradable hydrogels that progressively soften, mIOs maintain intermediate levels of YAP activity, and express markers similarly to those within Matrigel.^[^ ^244^ ^]^

Another approach is to engineer hydrogels such that enzymatic cleavage of peptide crosslinks by specific proteases allows for degradability. The susceptibility of peptide sequences to cleavage by specific mammalian proteases have been reported.^[^ ^259^ ^]^ This allows for the tailoring of hydrogels so that they can be remodeled by specific cell populations. For example, hydrogels have been formed that were crosslinked by either a peptide generally permissive for MMP degradation or by a peptide specifically cleaved by MMP9 or MMP14.^[^ ^260^ ^]^ The authors encapsulated fibroblasts and vascular smooth muscle cells and showed that while both grew to an equal extent within hydrogels generally permissive to MMP degradation, the smooth muscle cells proliferated two‐fold more and fibroblasts grew two‐fold less within the MMP14‐selective hydrogel.^[^ ^260^ ^]^ This demonstrated that it is possible to promote cell‐specific growth by tailoring degradability to a protease expressed by a specific cell type. This finding was further supported by a study demonstrating that neovascularization within PEG hydrogels could be improved by incorporating MMP‐specific crosslinks versus general collagenase permissive crosslinks.^[^ ^261^ ^]^

#### 5.3.2 Modelling Physiological and Pathological Matrix Remodeling

Strategies that incorporate specific enzymatic degradability into hydrogels have been used to assess the contribution of matrix remodeling to the growth and stemness of neural progenitor cells (NPCs). NPCs were embedded within ELP hydrogels with an elastin‐like domain and an adhesive bioactive domain susceptible to proteolysis.^[^ ^262^ ^]^ These materials were then exploited to show that expression of NPC stemness markers were increased in more degradable hydrogels yet remained insensitive to changes in stiffness. The authors further showed that hydrogels engineered to be susceptible to degradation by ADAM9, which is expressed by NPC, drove stemness maintenance. Thus, such systems may be suitable for limiting or promoting the growth of specific cell populations in cocultures, and could be applied to limit or enhance the growth of mesenchymal populations in models of the intestine.

Synthetic hydrogels have also been used to investigate ECM remodeling around HIOs to create models of CD fibrosis.^[^ ^177^ ^]^ PEG hydrogels are often formed using Michael additions between homo‐bifunctional cysteine residue terminating‐peptides with functionalized multi‐arm PEGs. However, at low polymer concentrations, this design can favor the formation of primary loops that reduce the number of PEG macromer arms available for hydrogel crosslinking, which can impact gelation kinetics. To overcome this issue, hydrogels were formed using a 2‐step process that relied on hetero‐bifunctional peptides to ensure that each could only react in a desired manner.^[^ ^177^, ^237^ ^]^ In these fully synthetic systems, co‐culture with ILC1 prompted HIO mesenchymal cells to remodel their surroundings through a combination of MMP‐mediated degradation and ILC1‐induced ECM deposition,^[^ ^177^, ^263^ ^]^ which is reminiscent of pathological matrix remodeling in patients with CD. In short, synthetic hydrogels that allow for ECM remodeling are suitable for studying physiological processes, and can be used to disentangle the relationship between mechanics and matrix remodeling in disease.

### 5.4 Engineering Tissue‐like Structures and Functions

Microfluidic or organ‐on‐chip approaches encompass strategies that use microchip‐based systems combined with microfluidic tubing to create tissue models. Chips can be designed to mimic tissue‐tissue interfaces and mimic physiological complexities that cannot be achieved using traditional cell culture approaches, making them ideal to replicate the complex, multi‐cellular interactions inherent in the native intestine.

#### 5.4.1 Cell–Cell Interactions

**Figure 6:**
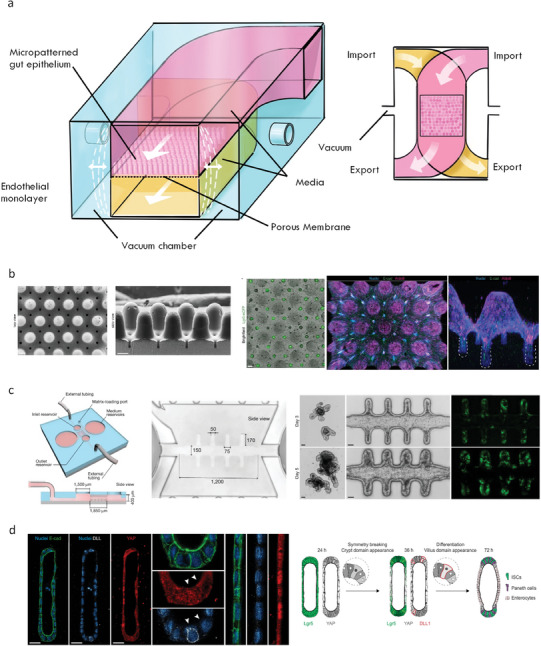
Examples of bioengineered intestinal models. a) Design of an intestine chip containing two parallel culture channels separated by a porous membrane lined on both sides by cells. Inlet and outlet channels allow for the addition of drugs or microbes, enabling independent analyses on each monolayer. Vacuum chambers on either side stretch the flexible membrane and adherent cells, mimicking peristalsis. b) (left) SEM images of PDMS stamp with crypt‐villus structures. (right) A hydrogel formed with the stamp and seeded with Lgr5‐GFP mIO and stained for E‐cadherin and AldoB. Lgr5+ ISCs localize to the crypts, while AldoB‐expressing enterocytes cover the villus. c) Design of cylindrical intestine chip. (left) The design consists of a central chamber containing a hydrogel flanked by inlet and outlet channels for luminal perfusion, and two lateral reservoirs supplying media and growth factors. (middle) Prior to cell loading, the hydrogel is laser ablated to construct a cylindrical channel with micro‐cavities resembling crypts. (right) The tubular scaffold is populated with cells to establish a confluent epithelium. d) (left) mIO cultured in pill‐shaped cavities and stained for E‐cadherin, YAP, and notch ligand (DLL) show differential expression in elongated versus restricted cells along the sides versus bases of the pill shape. (right) Proposed mechanism of geometry‐driven crypt specification. Panels (b) and (d) are reproduced with permission.^[^ ^269^ ^]^ 2022, The American Association for the Advancement of Science. Panel (c) is reproduced with permission.^[^ ^204^ ^]^ 2020, Springer Nature.

Double‐channel organ‐chips contain two parallel culture channels separated by an ECM‐coated porous membrane that can be lined on both sides by cells (**Figure** **6**a).^[^ ^187^, ^264^ ^]^ Exclusive inlet and outlet channels allow for inoculation with cells, compounds, or microbes, and for study of the cell monolayers independently. Surrounding the main channels are vacuum chambers through which cyclic suction can stretch the flexible sidewalls, mimicking peristalsis.^[^ ^187^, ^264^ ^]^ By culturing Caco‐2 and HT‐29 epithelial cells within them, gut chips have been used to study cellular responses to cyclical stretching. For example, stretch was found to prompt monolayers to grow microvilli structures and adopt barrier function superior to that observed in standard Transwell models. Conversely, lack of epithelial deformation triggered commensal bacterial overgrowth, similar to that observed in patients with IBD.^[^ ^264^ ^]^ Similar organ‐chip designs have highlighted how mechanical deformation can promote *Shigella* invasion, suggesting that peristalsis and intestinal architecture are key parameters for *Shigella* infection.^[^ ^265^ ^]^

More recently, IOs have been incorporated into gut‐chip designs.^[^ ^266^ ^]^ IECs derived from hIOs were seeded onto the upper‐side of the membrane while human intestinal microvascular endothelial cells lined the underside. Here, the authors reported elongated villi structures with polarized epithelium containing specialized IECs. Moreover, they demonstrated strong barrier function, secretion of digestive enzymes, and that cells nearer the base were proliferative.^[^ ^266^ ^]^ Such organ‐chips are remarkably versatile, providing opportunities for fundamental research on tissue‐tissue interactions, with the potential for preclinical drug screening. Accessibility of the fluidic and pneumatic systems required to operate chips currently hinders widespread adoption; however, there is progress toward making them more accessible with open‐source material.^[^ ^267^ ^]^

#### 5.4.2 Accessing the Apical Surface of the IE

A drawback of the organ‐chip platform is that the physiological relevance of the porous membranes central to their design is questionable since it lacks crypt/villus morphology or native ECM complexity. However, IOs grow as cystic structures, which are similarly problematic, as the enclosed lumen traps dead cells and waste inside, rather than allowing it to be removed by luminal flow. This limits the lifespan of IOs and restricts access to the apical surface of the epithelium. Thus, studies that require longer lifespans or that aim to assess barrier function or microbial‐epithelial interactions are complicated. One strategy to gain apical access to IOs involves flipping their polarity so that the apical surface faces the exterior environment.^[^ ^153^ ^]^ This enables analysis of barrier function and investigation into microbe interactions. Alternatively, microinjection of microbes and compounds into the lumen can be used; however, this is technically challenging, difficult to automate, and can damage IO.^[^ ^268^ ^]^

Bioengineering approaches have been applied to study fundamental processes along the epithelial apical surface, such as cell extrusion and shedding. To achieve this, a polydimethylsiloxane (PDMS) stamp formed with crypt‐villus‐like structures was used to template a hydrogel, onto which mIOs monolayers were seeded^[^ ^269^ ^]^ (Figure 6b). ISCs localized to the bottom of the crypt‐shaped indentations and differentiated IECs migrated up the villus‐like structures before being shed. Here, the engineered platform allowed for direct observation of the actin ring during extrusion, which plays a central role in maintaining barrier function, as well as analysis of the timing of apoptosis during the shedding process. Both of these fundamental processes are difficult to observe in in vivo models.

#### 5.4.3 Microfluidic Approaches to Generate Luminal Flow

To overcome issues associated with IO's growth as cystic structures, researchers have also developed approaches that combine apical access with luminal flow. For example, mIOs have been cultured within microfluidic chips lined with a hydrogel shaped to mimic intestinal morphology (Figure 6c).^[^ ^204^ ^]^ The core PDMS device consisted of a central chamber containing a hydrogel with inlet and outlet channels that allow for luminal perfusion, and lateral reservoirs, which supply media basally. Prior to cell loading, hydrogel in the central chamber was laser ablated to construct a cylindrical channel mimicking the native intestine's crypt‐villus structure. The resulting tubular scaffold was populated with mouse ISCs, which established a confluent epithelium. Whereas standard mIO lifespan is between 7–10 days, with regular perfusion, the lifespan of the system could be extended to a month or more.^[^ ^204^ ^]^ Within this device, cell fate patterning was reminiscent of that in the native tissue with crypt‐like regions containing ISCs and Paneth cells localized to the base of the micro‐cavities. By contrast, differentiated IECs including enterocytes and goblet cells were exclusively found in cells lining the lumen. Furthermore, proliferation was restricted to the crypt‐like regions while daughter cells migrated out to replenish differentiated cells, mimicking that seen in vivo.^[^ ^204^ ^]^ Using this system, the authors showed epithelial regeneration after dextran sodium sulfate cytotoxicity or low‐dose gamma radiation‐mediated damage. They also modelled parasitic infection with *Cryptosporidium parvum*, providing unprecedented insight into the in situ life cycle of the parasite and host‐microbe interactions.

**Figure 7:**
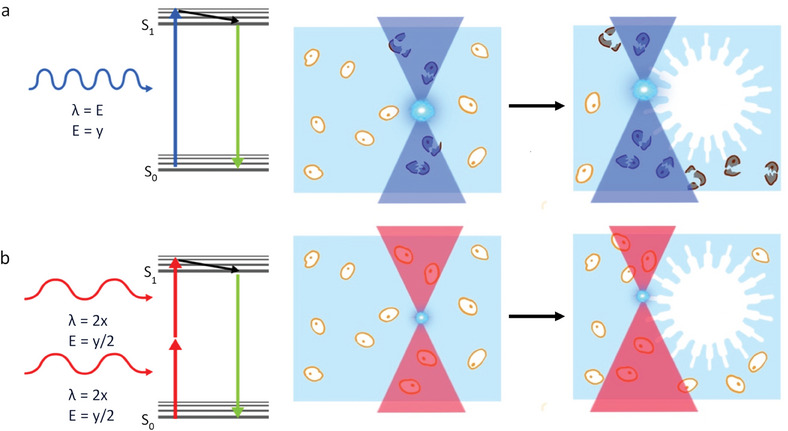
Schematic showing the potential of two photon microscopy to engineer an intestinal ECM. a) (left) One‐photon UV excitation and emission spectra. (right) Cross‐section of a synthetic hydrogel containing encapsulated cells designed to depolymerize in response to UV irradiation. The laser carves a topography through the hydrogel to create a 3D architecture reminiscent of the intestine. In this situation, encapsulated cells in the path of the laser light are negatively impacted. b) (left) Two‐photon infrared excitation and emission spectra. Two photon microscopy combines the energy of two colliding photons to equal that of UV irradiation. (right) Cross‐section of a synthetic hydrogel containing encapsulated cells designed to depolymerize in response to UV irradiation. Two‐photon laser depolymerization can carve an intestinal‐like topography, but here, encapsulated cells in the path of the laser are viable and the resolution of ablation is limited only by the optics.

Such strategies not only provide insight into how bioengineering may solve current limitations of tissue and disease models, but also offer avenues for future innovations. In creating a cylindrical channel, the authors used laser ablation to create a tissue‐mimicking structure, but this approach affords limited resolution and precludes real‐time structural editing in cell‐laden hydrogels. However, by combining advances in microscopy with tissue‐specific and user‐modulated hydrogel chemistries, this could be overcome. For example, photoresponsive hydrogel chemistries are available that support cell adhesion and remodeling, and offer user‐defined degradation by crosslinker photocleavage. Moreover, two‐photon microscopy using cytocompatible infrared lasers that combine at a single point can achieve UV wavelengths, avoiding damage across the optical path of the laser, while providing resolution limited only by optics (**Figure** **7**).^[^ ^270^ ^]^ Hence, combining two‐photon microscopy with UV‐cleavable hydrogel chemistries has the potential to provide real‐time 3D structural editing without compromising cells in situ.^[^ ^270^ ^]^

#### 5.4.4 Controlling Organoid Morphology and Niche Cues

As ISCs and Paneth cells localize to curved crypt‐like structures, bioengineering strategies have also been employed to ask fundamental questions about how physical cues drive crypt formation and cell fate specification. Pill‐shaped cavities were produced within ColI‐Matrigel hydrogels using soft lithography techniques and seeded with murine ISC^[^ ^269^ ^]^ (Figure 6d). Within these pill‐shaped structures, cells adopted elongated morphologies along the long walls and more restricted morphologies at the curved ends, resulting in preferential nuclear and cytoplasmic localization of YAP, respectively. Differential YAP signaling prompted localized notch signaling, thus establishing the location of the crypt. Hence, bioengineering approaches can complement *in viv* *o* approaches to reveal fundamental insight into processes such as crypt morphogenesis.

The mechanical properties of the intestine change over time both during development and in diseases like fibrosis. Moreover, the role of tissue mechanical cues in driving normal biological processes, like IE crypt formation, have recently been reported.^[^ ^269^ ^]^ Therefore, researchers have begun to incorporate on‐demand and site‐specific modulation of 3D hydrogel mechanical properties into their tissue models. Indeed, photo‐controlled chemistries are available that allow for user‐controlled photodegradation and photocross‐linking to locally soften or stiffen the microenvironment around encapsulated cells.^[^ ^271^, ^272^ ^]^ For example, hydrogels modified with *o*‐nitrobenzyl‐acrylate and methacrylate groups have been reported that enable photocleavage and photocross‐linking at distinct excitation wavelengths, respectively.^[^ ^272^ ^]^ Such photosensitive chemistries have been applied to mIO cultures. For example, a PEG‐Allyl sulphide‐based hydrogel that offers spatiotemporal control over degradation kinetics^[^ ^273^ ^]^ was shown to support both colony formation and mIO maturation from single ISCs.^[^ ^274^ ^]^ More recently, localized photopatterning of hydrogel mechanics and microtopology has allowed for user‐defined control over mIO morphology,^[^ ^269^ ^]^ confirming previous findings that heterogenous YAP expression predicted the location of Paneth cell differentiation.^[^ ^199^ ^]^ Thus, controlled chemistries can be used to direct epithelial patterning in the absence of biochemical cues. Although reversable photosensitive chemistries are yet to be adapted for gut models, technologies may soon be available and have the potential to allow for investigations into how mechanical cues associated with ECM remodeling impact intestinal biology.

In addition to changes in mechanical properties, the composition of the native intestinal ECM and its tethered signaling ligands also change. Various hydrogel chemistries allow for the inclusion and removal of signaling ligands, and present the possibility for dynamic decoration of hydrogels.^[^ ^275^ ^]^ For example, multiple rounds of protein photorelease in concert with photomediated protein ligation have been used to create complex interconnected protein patterning within hydrogels.^[^ ^276^ ^]^ Using this approach, epidermal cell‐laden hydrogels patterned with EGF allowed demonstration of photorelease‐mediated endocytosis of EGF by the epidermal cells.^[^ ^276^ ^]^ Such systems are still limited by issues with phototoxicity; however, this could be overcome by applying multiphoton microscopy, which can use near infrared femtosecond lasers to generate signals in the ultraviolet and visible range. This approach would enable the use of photochemistries designed for excitation at cytotoxic 365 nm using near infrared wavelengths. Although these chemistries are yet to be applied to intestinal models or IOs, they open avenues toward user‐defined photochemical matrix patterning^[^ ^270^, ^277^ ^]^ and directed IO symmetry breaking, and potentially even stem cell fate decisions using light‐induced chemistries.

## 6 Perspectives

In vitro models of the intestine that mimic the native tissue's multicellular complexity, architecture and composition offer tremendous promise for understanding basic physiological processes and untangling how they go awry in disease. The discovery more than a decade ago of IOs, which offer a convenient model of the complex intestinal epithelium, provide a compromise between cell lines and animal models, and have brought the possibility of in vitro tissue models to the forefront. To achieve better native tissue‐like fidelity, researchers are striving to co‐culture IOs with other enteric cell populations and incorporate additional soluble and non‐soluble cues. However, IOs remain reductionist models with important drawbacks that still need to be overcome before the potential of true tissue models can be realized. Recapitulating the physical structure of the intestine, including its mechanical properties and chemical complexity similarly remains a work in progress. However, synthetic hydrogels, which mimic many properties of the native ECM, and which can be engineered with controlled physical and biochemical properties that approximate those of the native tissue are bringing such tissue models closer to reality. Moreover, transcriptomic and proteomic approaches, including at the single cell level are advancing and will continue to allow for better understanding of the behavior of cells in in vitro models.

Imaging of 3D tissues and the cells within them is complicated, as many optical systems are optimized for use on thin 2D samples. One way to address this limitation is to plate and image 3D organoids as heterogenous monolayers that retain the capacity to reform 3D IOs.^[^ ^278^ ^]^ However, the advent of advanced tissue models has coincided with remarkable advances in 3D imaging. Non‐invasive optical sectioning using confocal or multiphoton microscopy and, more recently, light‐sheet fluorescence microscopy, have enabled imaging of fixed whole‐mount tissues in 3D. These approaches combined with advances in live imaging, optical clearing and machine learning, have enabled visualization of cell shape, cell fate decisions and cell‐cell interactions with exquisite detail.^[^ ^149^, ^279^, ^280^ ^]^ For example, an ingenious way to control the focal plane of 3D IOs without the need for custom built light‐sheet microscopes was recently reported. Termed a “triple decker sandwich” culture, mIOs were seeded within a coat of Matrigel onto a non‐adhesive PolyHEMA hydrogel and followed with a final layer of Matrigel on top. This not only enabled high‐quality imaging using confocal microscopy, but also improved mIO viability by homogenizing nutrient access.^[^ ^281^, ^282^ ^]^

Organoid technologies have provided unprecedented insight into intestinal biology and are bridging the gap between animal models and in vitro experimentation. Dissecting the chemical niche of the IE has enabled researchers to develop IOs with user‐defined cellular identity and advances in multi‐omics are providing insights into how each IEC type arises and contributes to physiology and disease. Coupled with the development of designer matrices with tunable chemical and mechanical properties, it is now possible to engineer physiological signals to guide ISC self‐organization and patterning in a manner more representative of physiology. Moreover, pioneering approaches that circumvent well‐recognized limitations of standard cell cultures are enabling the development of a new field of research that combines stem cell self‐organization with bioengineering. However, while IOs are a wonderful equalizer in research and an ideal tool for picking the abundant low hanging fruit, at present, many bioengineering technologies remain relatively inaccessible. Standardization of protocols to maintain physiological parameters, and cell populations with predictable identity, developmental stage, and spatial distribution, will enable advances toward scaled‐up high‐throughput experiments. Coupled with patient biobanks, there exists vast potential to model and deliver patient‐specific treatments for disease. Now is an incredibly exciting time for research to create advanced tissue models and the intestine is an organ at the forefront.

## Conflict of Interest

The authors declare no conflict of interest.
